# Estimating Air Temperature and Its Influence on Malaria Transmission across Africa

**DOI:** 10.1371/journal.pone.0056487

**Published:** 2013-02-20

**Authors:** Tini Garske, Neil M. Ferguson, Azra C. Ghani

**Affiliations:** MRC Centre for Outbreak Analysis and Modelling, Department of Infectious Disease Epidemiology, Imperial College London, London, United Kingdom; Tulane University School of Public Health and Tropical Medicine, United States of America

## Abstract

Malaria transmission is strongly influenced by climatic conditions which determine the abundance and seasonal dynamics of the *Anopheles* vector. In particular, water temperature influences larval development rates whereas air temperature determines adult longevity as well as the rate of parasite development within the adult mosquito. Although data on land surface temperature exist at a spatial resolution of approximately 1 km globally with four time steps per day, comparable data are not currently available for air temperature. In order to address this gap and demonstrate the importance of using the right type of temperature data, we fitted simple models of the relationship between land-surface and air temperature at lower resolution to obtain a high resolution estimate of air temperature across Africa. We then used these estimates to calculate some crucial malaria transmission parameters that strongly depend on air temperatures. Our results demonstrate substantial differences between air and surface temperatures that impact temperature-based maps of areas suitable for transmission. We present high resolution maps of the malaria transmission parameters driven by air temperature and their seasonal variation. The fitted air temperature datasets are made publicly available alongside this publication.

## Introduction

Malaria is one of the major health threats globally, causing an estimated 220 million cases and 660 thousand deaths annually, with 80% of the burden occurring in Africa alone [1]. The parasite is transmitted by the *Anopheles* mosquito, with over 70 *Anopheles* species able to act as efficient vectors [2]. Substantial improvements in control of the disease have been achieved in the past decade, primarily through distribution of long-lasting insecticide-treated nets (LLINs) and use of indoor residual spraying with insecticides (IRS) [1]. These interventions reduce mosquito abundance by directly killing adult females and through their repellent effect which results in an increase in the duration of the gonotrophic cycle [3]. However, if such controls are relaxed without underlying changes in the environment, vector abundance can be expected to return to pre-intervention levels unless the mosquito population is entirely removed. Thus understanding the environmental factors influencing the intrinsic potential for malaria transmission in the absence of control (i.e. the basic reproduction number, 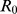) is important to determine appropriate strategies to further reduce transmission and move towards local elimination of the parasite.

Climate is one critical determinant of the suitability of geographical locations to support *Anopheles* habitats. Mosquito abundance is largely driven by rainfall patterns [4], [5], but is also dependent on temperature via complex influences on mosquito population dynamics, as well as on parasite development within the mosquito [6]. The duration of the aquatic stages of mosquito development has been shown to depend strongly on water temperatures in controlled laboratory experiments [7], [8], whereas the adult mosquito stages are dependent on ambient air temperatures via temperature-dependent survival and biting rates. Furthermore, parasite development within the mosquito is strongly temperature dependent, thereby influencing malaria transmission potential [6].

Two approaches have been employed in the literature to incorporate the temperature dependence of malaria transmission into models of climate suitability for malaria. Firstly, with regression modelling techniques using temperature (among other environmental data) as a covariate, empirical correlations can be established [9], [10], [11], [12], [13], [14], [15], [16], [17], [18]. Secondly, by using mechanistic equations of the temperature dependencies of the various stages, temperature can be incorporated into malaria transmission models [6], [19], [20], [21], [22], [23], [24], [25], [26]. The latter approach has the strength that it incorporates pre-existing understanding of the temperature dependencies, taking into account their highly non-linear nature. However, care should be taken in choosing the appropriate temperature variable as there are important differences between air and water temperatures [27], [28], indoor and outdoor air temperatures [29], as well as diurnal fluctuations in temperature that have been shown to influence disease dynamics [30], [31]. In earlier work, these subtleties were often ignored, with mean monthly air temperatures typically being used [22], [24], [26], sometimes even to model the influence of temperature on both aquatic and adult mosquito stages [6], [19], [25]. In more recent work, diurnal temperature variations have been incorporated by superimposing daily fluctuations between minimum and maximum temperatures [20], [31].

In recent years there have been substantial advances in mapping the geographic distribution of malaria and its vector species, driven by the availability of increasing amounts of computing power required for geostatistical modelling and analysis and by the availability of remotely-sensed climatic datasets [11], [15], [20], [32], [33], [34], [35], [36]. In particular the MODIS (MODerate resolution Imaging Spectroradiometer) project has been a rich source of climatic data, providing daily measurements of land surface temperatures at a spatial resolution of approximately 1 km globally since the year 2000 [37]. However, for the adult mosquito life cycle, ambient air temperature is more relevant. Another source of spatial data is the ERA project, which assimilates and reanalyses global climate data in order to produce estimates of a large number of atmospheric quantities, among them air temperatures. However, whilst these are provided at high temporal resolution, the spatial resolution is fairly low (0.75°, or approximately 75 km) [38]. To our knowledge there are no higher resolution data sources for air temperatures that have a shorter than monthly timestep.

The aim of this study was to fill this data gap and generate estimates of air temperatures for the African continent at a spatial resolution of 1 km, and to use this dataset to calculate the several crucial malaria transmission parameters that depend on air temperatures. To this end, we fitted a linear multilevel regression model to the air temperatures provided in the ERA dataset, using datasets such as surface temperatures, vegetation indices and land cover classifications as potential covariates, which we then extrapolated to the higher spatial resolution at which these data are available. We then defined the temperature suitability index taking into account the mechanistic understanding of the temperature dependence of several processes governing the adult mosquito life cycle and, using the estimated air temperature dataset, calculated these quantities across Africa throughout the year accounting for diurnal [31] and seasonal temperature variation.

## Methods

### Datasets and Data Preparation

#### Air temperature

The ERA Interim analysis [38] of the data of the air temperature 2 m above ground level for Africa was downloaded on a spatial grid of 1.5° by 1.5° resolution [39]. The 1113 grid locations which fall on the land mass of the African continent were selected for inclusion in our model. 2 m air temperature estimates were available at 0, 6, 12 and 18 UCT (coordinated universal time) each day, and the lowest and highest value for each day were selected to represent the minimum and maximum daily temperatures for each location. Note that while the dataset was also available at a higher resolution 0.75°, we would not have been able to perform the model fitting with the size of this dataset due to computational constraints.

#### Covariates

The MODIS instruments on the Terra and Aqua satellites view the entire earth’s surface every 1 or 2 days and take measurements in 36 spectral bands which are processed to produce a range of products. We obtained data on land surface temperature for day and night time [40], enhanced vegetation index (EVI), middle infrared reflectance (MIR) [41] and land cover classifications [42], all on a sinusoidal projection at approximately 1 km spatial resolution.

Eight-day aggregate time series of land surface temperature, containing measurements of both day and night time temperature, are produced by both Terra and Aqua, which cross any given location at different times of the day (products MOD11A2 and MYD11A2). To approximate the minimum and maximum daily temperatures, we combined the time series from Terra and Aqua by selecting the maximum day time and the minimum night time measurement for each time point and location. Measurements of EVI and MIR are available as 16-day time series from both Terra and Aqua (products MOD13A2 and MYD13A2), but are offset against each other by 8 days, such that they can be combined to yield 8-day time series. The IGBP global vegetation classification scheme from Terra (MOD12Q1) was obtained for the year 2004, which identifies 17 land cover classes [43].

Daily time series of estimated precipitation were obtained from the RFE2 dataset produced by the US National Oceanic and Atmospheric Administration [44]. These data are available on a 0.1° (∼10 km) spatial resolution.

Altitude data at 30″ ( = 1/120°, or approximately 1 km) resolution (albeit on a different grid than the MODIS data) were obtained from WorldClim [45], [46].

#### Temporal and spatial smoothing

We obtained time series of the day and night time air and surface temperatures, EVI, MIR and rainfall datasets for the years 2003 to 2006. To achieve the same temporal resolution for all datasets and to prepare data for the Fourier transforms, we aggregated the daily time series of air temperature and rainfall to a time step of 5.71 days, yielding 256 time points over the 4 years, whereas we interpolated the surface temperatures, EVI and MIR, which were obtained at 8 day resolution, to the same resolution of 5.71 days. This re-sampling avoids problems with recovering exact annual frequencies in the Fourier transforms [47]. For further details about the Fourier transforms and the impact of missing data see Text S1.

Data from all datasets at approximately 1 km spatial resolution (all MODIS datasets and altitude) were averaged within a radius of 5 km around each location to provide a spatially smooth surface. A sensitivity analysis for the choice of radius is presented in Text S1. For interpolation to a higher resolution than the 0.1° resolution on which the rainfall data were available, we used rainfall data from the closest grid point of the 0.1° grid for each location.

### Estimation of Air Temperature from Land-surface Temperature

To estimate air temperatures we fitted a hierarchical linear regression model [48] to the air temperature estimates from the ERA dataset, where the observations for individual time points (level one) are nested within locations (level two), i.e., for each location there are several observations at different time points.

In the model fitting, we employed a fairly exhaustive variable selection procedure to select the best model. With the size of the dataset fitted to and the large number of potential covariates fitting all possible model structures in the full hierarchical model framework was not computationally feasible. Instead, we broke the procedure down into several steps, each of which less computationally demanding [48].

Let 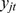 denote the air temperature at location 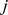 at time 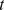. To predict this we utilise both time-varying and non-time-varying covariates (see Tables 1 and 2). Let 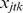 denote covariates that vary over time, where 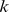 indexes the covariates, and 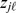 denote the covariates that do not vary by time where 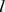 is an index for the covariates.

**Table 1:** Coefficients (95% CIs) of time series and location based variables, interactions between these as well as variances of the random effects for the model fitted to night time air temperatures.

	regression coefficients	Interaction terms with time series variables				
		night surface temperature	day surface temperature	EVI	MIR	rainfall
Intercept	60.5 (56–65.1)	x	x	x	x	x
night surface temperature	−7.2 (−7.9–−6.5)	x	x	x	x	x
day surface temperature	3.51 (3.16–3.86)	x	x	x	x	x
EVI	−4.4 (−5.6–−3.1)	x	x	x	x	x
MIR	−8.4 (−10–−6.9)	x	x	x	x	x
rainfall	−0.5 (−0.85–−0.15)	x	x	x	x	x
lat	1.41 (1.01–1.81)	–	−0.19 (−0.25–−0.13)	−0.42 (−0.57–−0.27)	0.54 (0.38–0.7)	–
lon	−0.15 (−0.49–0.19)	−0.37 (−0.43–−0.32)	0.244 (0.195–0.292)	0.29 (0.17–0.41)	−0.3 (−0.43–−0.17)	–
abs(lat)	−7.4 (−8–−6.8)	1.26 (1.15–1.36)	–	0.7 (0.51–0.89)	0.91 (0.67–1.15)	0.243 (0.2–0.286)
mean night surface temperature	−3.4 (−4–−2.7)	0.7 (0.57–0.83)	−0.53 (−0.59–−0.47)	0.32 (0.18–0.45)	1.36 (1.09–1.62)	0.106 (0.06–0.152)
mean day surface temperature	−2.95 (−3.42–−2.48)	0.7 (0.6–0.79)	–	–	0.51 (0.32–0.69)	–
mean EVI	−1.7 (−2.3–−1.2)	–	–	0.56 (0.33–0.8)	–	–
mean MIR	−4.6 (−5.06–−4.14)	0.56 (0.47–0.65)	–	1.63 (1.36–1.9)	–	−0.21 (−0.26–−0.15)
mean rainfall	0.32 (0.04–0.59)	–	–	–	–	−0.098 (−0.138–−0.057)
altitude	−3.5 (−3.92–−3.08)	0.69 (0.6–0.78)	−0.34 (−0.4–−0.28)	–	0.88 (0.7–1.07)	0.124 (0.089–0.158)
evergreen broadleaf forest	−0.19 (−0.5–0.11)	0.08 (0.02–0.15)	–	–	−0.4 (−0.54–−0.26)	0.048 (0.026–0.07)
deciduous broadleaf forest	−0.24 (−0.41–−0.06)	0.134 (0.087–0.182)	–	–	–	−0.034 (−0.052–−0.017)
closed shrubland	–	–	–	–	–	–
open shrublands	2.34 (1.95–2.72)	−0.08 (−0.13–−0.03)	−0.165 (−0.208–−0.123)	−0.69 (−0.82–−0.55)	−0.63 (−0.76–−0.49)	–
woody savannas	–	–	–	–	–	–
savannas	–	–	–	–	–	–
grasslands	−0.36 (−0.51–−0.22)	–	–	–	–	0.038 (0.017–0.059)
croplands	0.13 (−0.03–0.3)	–	−0.099 (−0.138–−0.06)	–	–	–
barren or sparsely vegetated	3 (2.3–3.6)	–	−0.26 (−0.33–−0.2)	−2.03 (−2.31–−1.75)	−0.75 (−0.97–−0.53)	0.399 (0.35–0.449)
variance of random effects	17	0.57	0.36	1.6	2	0.045

x indicates interaction terms not considered.

– indicates variables not included in the final model.

**Table 2:** Coefficients of time series and location based variables, interactions between these as well as variances of the random effects for the model fitted to day time air temperatures.

	regression coefficients	Interaction terms with time series variables				
		night surface temperature	day surface temperature	EVI	MIR	rainfall
Intercept	41.1 (36.6–45.7)	x	x	x	x	x
night surface temperature	−2.4 (−3.1–−1.6)	x	x	x	x	x
day surface temperature	2.44 (2.05–2.83)	x	x	x	x	x
EVI	−1 (−2.3–0.4)	x	x	x	x	x
MIR	−7.5 (−8.8–−6.3)	x	x	x	x	x
rainfall	−0.73 (−1.07–−0.39)	x	x	x	x	x
lat	0.2 (−0.24–0.64)	0.2 (0.13–0.26)	–	−0.17 (−0.3–−0.05)	0.46 (0.29–0.63)	–
lon	0.09 (−0.28–0.47)	−0.33 (−0.39–−0.27)	0.152 (0.104–0.2)	0.18 (0.08–0.28)	−0.33 (−0.47–−0.19)	–
abs(lat)	−5 (−5.5–−4.5)	0.83 (0.73–0.93)	0.36 (0.28–0.44)	0.44 (0.27–0.62)	–	0.14 (0.09–0.19)
mean night surface temperature	−3.8 (−4.5–−3.1)	0.48 (0.37–0.6)	−0.17 (−0.24–−0.11)	0.55 (0.33–0.77)	1.12 (0.94–1.31)	0.16 (0.1–0.22)
mean day surface temperature	0.5 (−0.1–1)	0.15 (0.06–0.24)	–	−0.63 (−0.79–−0.47)	0.38 (0.19–0.57)	−0.121 (−0.155–−0.086)
mean EVI	–	–	–	–	–	–
mean MIR	−3.3 (−3.8–−2.8)	–	0.42 (0.32–0.51)	1.03 (0.8–1.27)	–	–
mean rainfall	1.6 (1.1–2.2)	−0.25 (−0.36–−0.14)	–	−0.22 (−0.37–−0.06)	–	–
altitude	−3.3 (−3.8–−2.8)	0.42 (0.34–0.5)	–	0.22 (0.07–0.38)	0.75 (0.57–0.92)	0.077 (0.035–0.119)
evergreen broadleaf forest	0.28 (−0.02–0.58)	–	–	–	−0.68 (−0.84–−0.51)	–
deciduous broadleaf forest	0.28 (0.16–0.39)	–	–	–	–	−0.032 (−0.053–−0.012)
closed shrubland	–	–	–	–	–	–
open shrublands	1.54 (1.24–1.84)	–	−0.28 (−0.34–−0.23)	−0.29 (−0.39–−0.19)	–	−0.093 (−0.12–−0.065)
woody savannas	1.07 (0.77–1.38)	−0.13 (−0.19–−0.08)	–	−0.14 (−0.22–−0.06)	–	–
savannas	−0.01 (−0.26–0.23)	–	–	0.23 (0.14–0.32)	–	–
grasslands	–	–	–	–	–	–
croplands	–	–	–	–	–	–
barren or sparsely vegetated	−0.4 (−1–0.2)	0.36 (0.27–0.44)	−0.57 (−0.67–−0.47)	–	0.98 (0.77–1.19)	0.124 (0.08–0.167)
variance of random effects	19	0.49	0.42	0.75	2.2	0.063

x indicates interaction terms not considered.

– indicates variables not included in the final model.

Firstly we fitted a model only including the time-series variables and their random slopes,
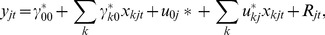
(1)where 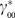 and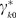 are the fixed effect parameters, 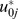 are random intercepts, 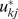 are random slopes associated with the time-series variables 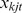 and 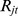 is the error term.

We fitted models that include all possible combinations of the time series variables with and without allowing for a random slope for each of the included variables. Let 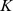 be the number of time series variables available (here, 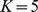), and 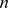 the number of time series variables included in a model. There are 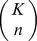 different variable combinations in which 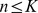 variables can be included, and 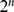 models for each of these combinations, as for each variable a random slope can be included or not. This gives 243 different models for 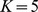 time series variables. Note that all of these models included a random intercept. Of these models, the best was selected based on the Bayesian Information Criterion (BIC) [49]. For the random intercept and each random slope included in the best model, we obtained estimates of the random effects 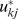, 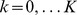 at each location.

Next, in order to explain the random effects, we fitted ordinary linear models to the estimates of each of the random effects, using all the fixed effect covariates as potential explanatory variables:
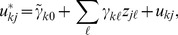
(2)where 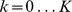. Here 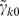 is the intercept, 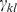 are the slope parameters and 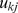 is the component of the random intercept and slopes that cannot be explained by the static covariates 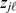. While 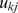 is technically an error term in equation (2), this very parameter is the random slope in the full model after inserting this back into equation (1) to obtain the final model. For variable selection, we fitted all 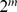 possible combination of variables (where 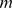 is the number of potential static covariates 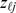, 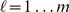) and selected the best model based on the BIC for each 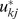. Inserting equation (2) into equation (1) returns the final model:
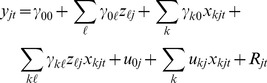
(3)where



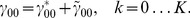
(4)While this 2 step approach yields the same model structure as could be obtained using the full hierarchical model from the start, estimating the parameters in the 2 step approach is not statistically efficient, and therefore, we finally re-fitted the full model (3) in a single-step, including only the variables and interaction terms identified in steps 1 and 2. In order to assess if the level-two variables 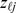 explain the step-1 random effects 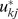 sufficiently, such that the remainder 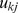 was small, we also fitted the full model, but with each random slope excluded separately and performed a deviance test to assess significance of the excluded random slope.

Model fitting was undertaken using the lme4 package in the R software version 2.13.0.

### Model Validation and Extrapolation to Higher Resolution

To validate the model fit, we re-fitted the models to a validation dataset containing 90% of the data (excluding the full time series of a randomly chosen 10%). We then extrapolated the validation model to the excluded locations to obtain estimates of the air temperature at these locations by using the parameter estimates obtained from the validation model in conjunction with the values of the covariates at the locations excluded in this fit. We compared the obtained air temperature estimates with the input data as well as assessing the model fit to the full dataset.

A complication for applying the model to non-fitted locations was that the random effects were only estimated for the locations the model was fitted to, so they needed to be extrapolated to any further locations of interest first. To achieve this, we used ordinary kriging to make use of the spatial correlations between the estimated random effects [50]. To this end, we fitted functional forms of the exponential type to the observed variograms, described by
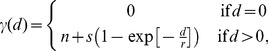
(5)where 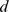 is the distance (here measured in ° latitude/longitude), 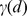 is the semivariance at distance 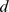, and 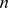, 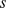 and 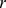 are the nugget, sill and range parameters of the model, respectively. We then used these fitted variograms in the kriging. Briefly, the random effect extrapolated to any new location 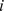, 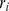 is given by a linear combination of the random effects 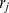 at the grid locations 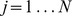, 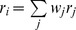. The weights 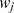 are determined by
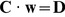
(6)with 
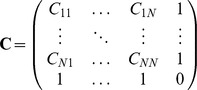
,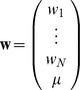 and 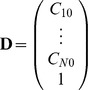.

The 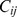 are the covariances between locations 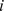 and 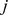, as determined by their distance 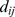 and the variogram 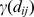 as 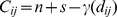. The Lagrange parameter 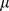 is used to ensure the estimate is unbiased via the condition 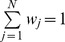; it can also be used to estimate the error variance.

We used the same process, but with the final model fitted to the full dataset, to extrapolate the model to the higher resolution at which the covariates were available. Finally we used a Fast Fourier Transform algorithm [51] to perform Fourier transforms of the observed and estimated air temperatures as well as the covariate time series, and reconstructed time series based on the constant term and the annual and biannual frequencies to represent smoothed overall seasonality trends. Further details of the Fourier transforms and the identification of relevant frequencies are given in Text S1.

### Malaria Transmission Indices

In the Ross-Macdonald model for malaria transmission [52], the basic reproduction number for malaria is given by
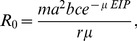
(7)where 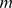 is the vector:host ratio, 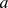 the mosquito biting rate, 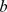 and 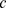 transmission probabilities from host to vector and vector to host, respectively, 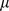 the mosquito mortality rate, 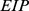 the extrinsic incubation period and 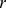 the recovery rate of the human host. Here, the term 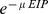 describes the probability that an infected mosquito will survive the incubation period, whereas 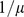 is the average duration of infectiousness given survival to the onset of infectiousness, such that the average infectious period of any mosquito is determined by 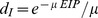. The average number of blood meals an infected mosquito takes during its infectious period is 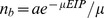. The dependence of 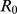 on air temperature can be summarised by the temperature dependence in this quantity (termed the temperature suitability index):
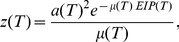
(8)(note that this definition differs slightly from that used in [20]).

To illustrate the effect that temperature variation can have on malaria transmission, we calculated these quantities across Africa at high resolution taking into account the diurnal cycles and seasonality in temperature. Whilst temperature could also influence the vector to host ratio 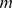, this is normally dominated by the dependence on rainfall. Furthermore any temperature dependence on mosquito population size is largely an effect of water temperatures governing the larval development and therefore not well represented by the air temperature dataset considered in this study.

#### Biting rate

The frequency of biting is largely determined by the time it takes the mosquito (i) to search for a host and attack, (i) to digest a blood meal, and (iii) to search for a suitable oviposition site. While the first and third phases together take approximately 24 h irrespective of temperature, the second phase exhibits strong temperature dependence, although data characterising this dependence is sparse in the recent literature. However, data for *An. maculipennis*, a vector similar to Anopheles gambiae [53], at a relative humidity of 70 to 80% suggests a biting rate dependence on temperature of
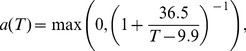
(9)where temperature 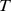 is measured in °C and the time unit is days [21], [54], [55]. For simplicity, we neglect the presence of non-human potential hosts and assume that all bites are on humans.

#### Extrinsic incubation period

Once the parasite has entered the mosquito, it needs to complete a number of developmental stages before it can be transmitted on to a human host. To calculate the extrinsic incubation period of *Plasmodium falciparum* in the mosquito, we use a widely used linear functional form for the dependence of the parasite development rate on temperature, 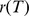 [6]

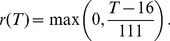
(10)


The extrinsic incubation period 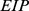
can then be determined by solving the integral
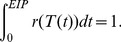
(11)


#### Extrinsic infectious period

Assuming that mosquitoes experience a temperature-dependent death rate, 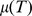 given by
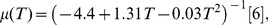
(12)the probability that a mosquito survives the incubation period is given by



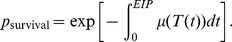
(13)The remaining life expectancy 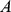 following onset of infectiousness, given that the mosquito has survived the extrinsic incubation period 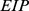, is then
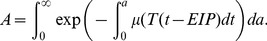
(14)


The mean duration of infectiousness of mosquitoes, termed extrinsic infectious period, is therefore given by 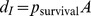.

#### Number of infectious bites and temperature suitability index

The number of infectious bites per infected mosquito 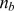 is defined as the product of the duration of the infectious period and the daily average biting rate at the time of the onset of infectiousness, a fairly accurate approximation given that the typical duration of infectiousness is only a few days and the linear dependence of the biting rate on temperature. In order to calculate the temperature suitability index 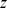 we multiply this by the biting rate at the time of infection of the mosquito.

#### Diurnal temperature curves

To calculate both extrinsic incubation and infectious periods, we use temperature curves that take into account the diurnal variations as well as the seasonal changes. The diurnal temperature cycle is modelled according to a sine wave during day time, and exponential decay at night [31], [56] as
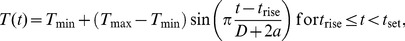
(15)

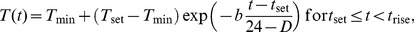
(16)where 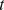 is the time of day, 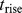 and 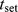 are the times of sunrise and sunset.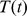 is the temperature at time 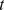, and 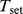 is the temperature at sunset. 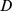 is the number of hours of daylight, and 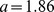 and 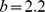 are model parameters as fitted by Parton and Logan [56]. We use the temperature measurement during the day, 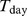, for 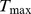, whereas 
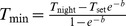
 such that the lowest temperature reached in the diurnal curve is the night-time temperature measurement, 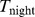. Sunrise and sunset times for each day of the year and location are calculated according to [57], [58]. Temperature curves are constructed in hourly intervals over the year for each location, using the smoothed day and night time temperatures obtained from the Fourier transforms for modelled air temperatures including the constant term as well as the annual and biannual modes.

In our calculations, mosquitoes are infected each day at midnight. For each of these mosquitoes the duration of the extrinsic incubation period, the probability of survival and the life expectancy after the onset of infectiousness are calculated based on the hourly temperature curves for any given location and season. For computational purposes we incorporate a maximum mosquito life span of 60 days.

## Results

### Relationship between Land Surface and Air Temperature

**Figure 1:**
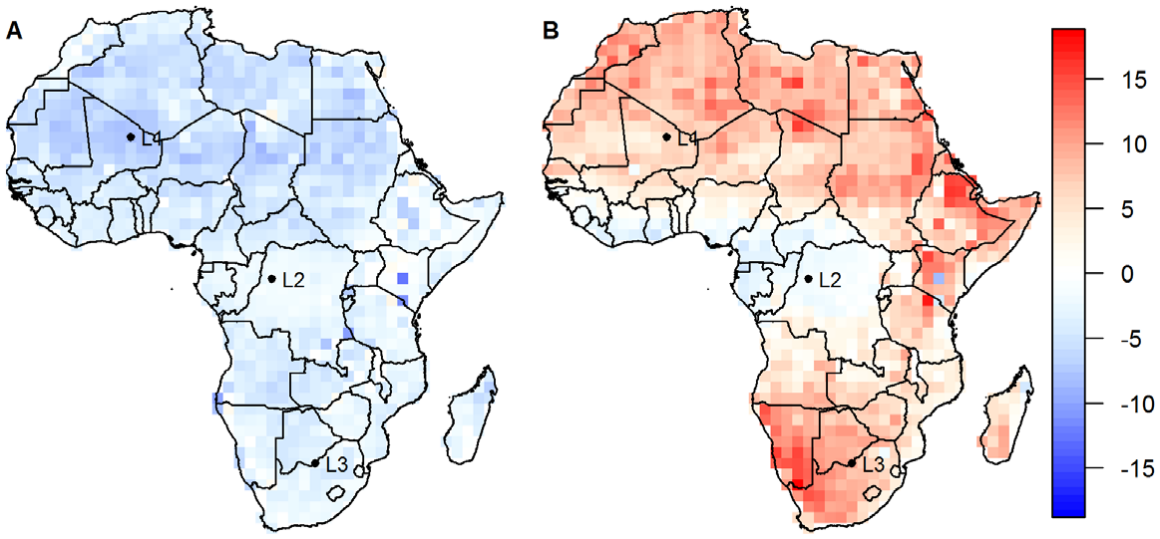
Annual mean differences between observed surface and air temperature during night (left) and day (right). Locations L1, L2 and L3 at latitudes 19.5, 0, and −25.5 and longitudes 0. 19.5 and 25.5, respectively, give the locations for the time series shown in Figure 2.

**Figure 2:**
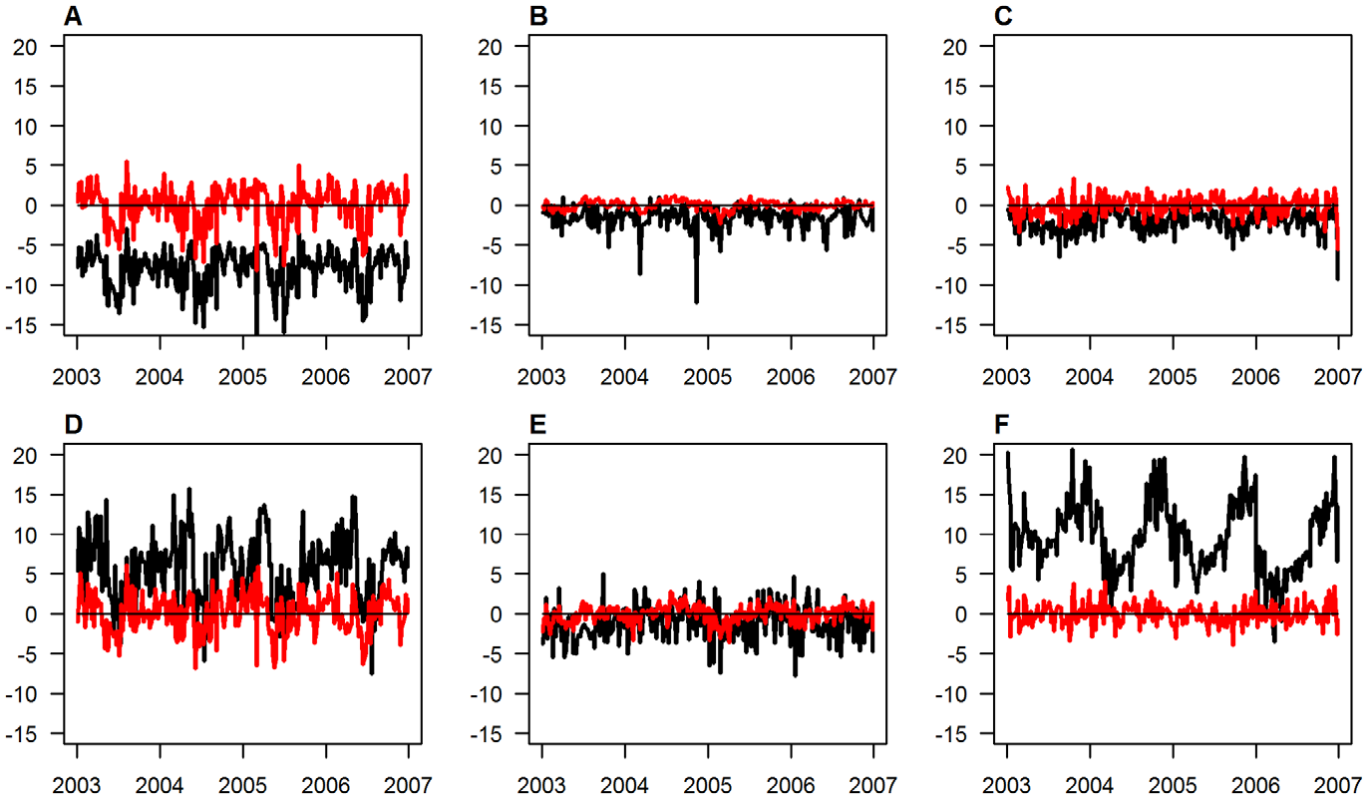
Differences between surface and air temperatures over time for three selected locations. Locations as marked in Figure 1: L1 left (panels A and D), L2 middle (panels B and E) and L3 right (panels C and F), respectively, for night time (top: panels A, B, C) and day time (bottom: panels D, E, F). Red lines for difference between fitted and air temperatures, black lines for difference between surface and air temperatures.

The observed air temperatures differ from the corresponding land surface temperatures in the same location in a complex manner. During the night, mean surface temperatures across the year are below the corresponding mean air temperatures, whereas during the day, they tend to be considerably higher than the corresponding mean air temperatures in most locations apart from parts of central western Africa (Figure 1). Furthermore, there is additional variation in the difference between air and surface temperatures over calendar time (Figure 2). For example, in Figure 2F (which refers to location L3 in Figure 1B), the observed surface temperatures are higher than the observed air temperatures throughout the year, but the difference between surface and air temperatures is much higher during summer than during winter.

The best fitting model fitted to both the night and day air temperature time series included night and day surface temperature, EVI, MIR and rainfall as time series and latitude, longitude, altitude and various land cover classifications as static variables as well as interaction terms between the time series and static variables. A random slope and random effects for all time series variables were also included (Tables 1 and 2).

**Figure 3:**
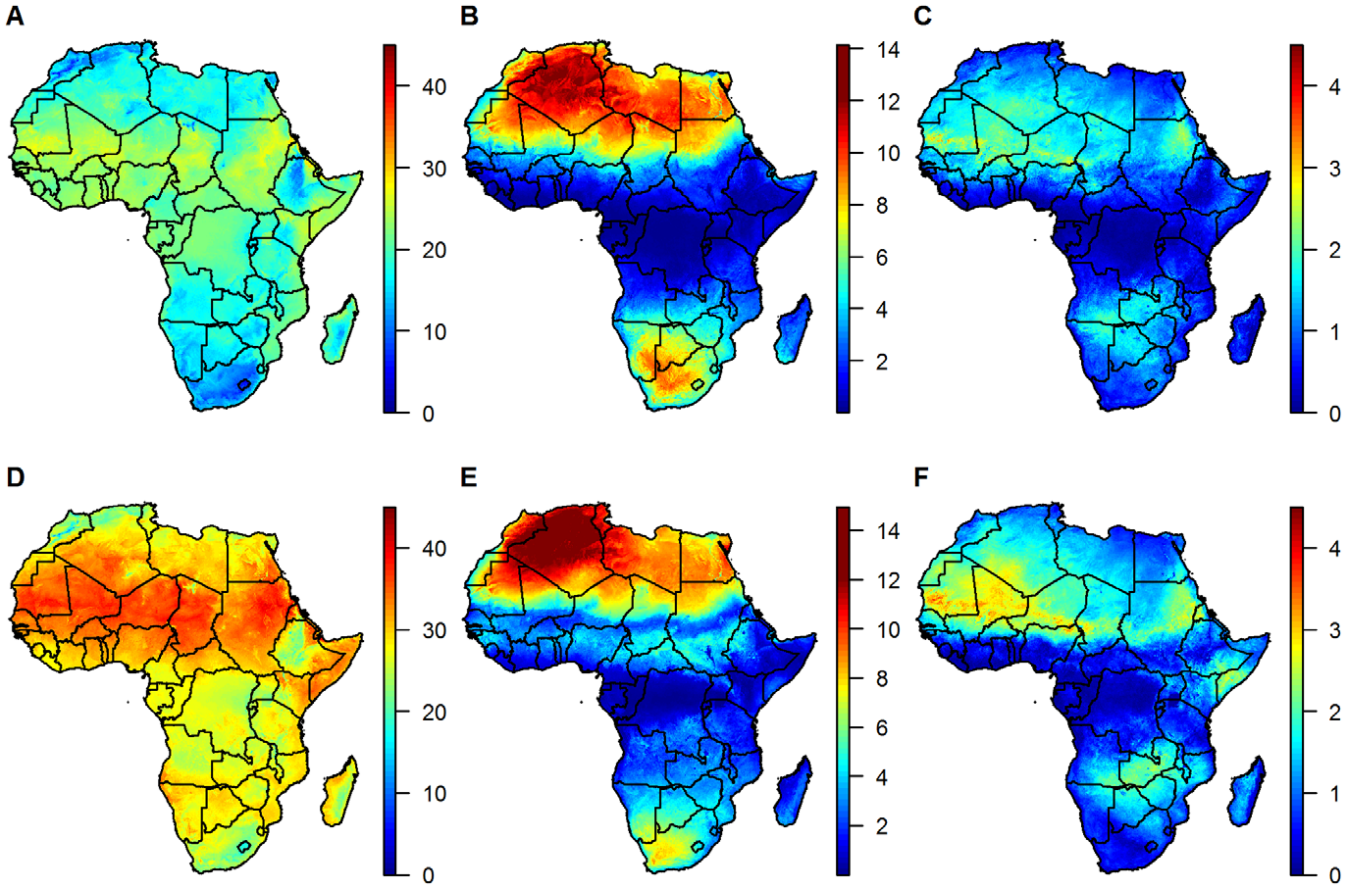
Fitted air temperature in °C extrapolated to a 0.1° grid. Mean temperatures (A and D) as well as the amplitudes of the annual (B and E) and biannual (C and F) modes of the Fourier transform for night (top) and day time (bottom), respectively.

The annual mean of the estimated air temperatures reproduced the observed air temperatures near perfectly, and the correlations between the observed and estimated air temperature time series were much higher than those between the observed air and surface temperatures (Table 3). This could also be seen in the time series of the differences between estimated and observed air temperatures (red lines in Figure 2), which show fast fluctuations around the mean value of 0, but little seasonal variation, demonstrating that the seasonal patterns were captured very well by our model. It is exactly these seasonal patterns that were captured in the Fourier transforms, which show annual amplitudes of temperature variation close to 0 in the equatorial region, increasing to around 10 in southern and up to 14°C in northern Africa, with biannual amplitudes generally considerably smaller apart from some areas in eastern Africa where the biannual modes were stronger than the annual modes (Figure 3).

**Table 3:** Temperature ranges and correlations between air and surface temperatures across Africa.

	median (95% range) of temperature in °Celsius	correlation with air temperature (95% CI)	correlation of the mean over the year with mean air temperature (95% CI)
night air temperature	20.8 (6.0–29.5)	–	–
night surface temperature	17.5 (1.7–24.4)	0.881 (0.880–0.882)	0.84 (0.82–0.86)
fitted night air temperature	20.9 (6.3–29.1)	0.967 (0.967–0.967)	1.00 (1.00–1.00)
day air temperature	29.2 (17.0–41.3)	–	–
day surface temperature	34.6 (19.8–52.0)	0.778 (0.777–0.780)	0.70 (0.67–0.72)
fitted day air temperature	29.1 (17.5–40.9)	0.961 (0.961–0.961)	1.00 (1.00–1.00)

Data are based on the time series between 2003 and 2006 aggregated to 64 time points per year, showing correlations (95% confidence intervals based on 1000 bootstrap samples) between time series and between means of the time series for night and day time.

– for correlations of a dataset with itself.

### Model Validation

For model validation we compared the output of the model fitted to the full dataset at 1113 locations on a 1.5° grid to that of the validation model fitted to the data from 90% of the locations in the full dataset. Variograms of the random effects obtained by fitting to the full dataset are very similar to those obtained by fitting to the validation dataset (see Table S1 as well as Figures S11 and S12 in Text S1).

We extrapolated the validation model to those locations that were excluded (using the variograms obtained from the validation model) and compared the model output from the full model at these locations with the extrapolation obtained from the validation model to assess the magnitude of the errors made in the extrapolation process. The correlations between the random effects of the full and extrapolated validation model are positive throughout, but variable in magnitude (see Table 4 and Figures S13 and S14 in Text S1). The variances of the random effects estimated in the full model at the locations excluded in the validation dataset were considerably larger than those extrapolated from the validation model (see Table 5). These results demonstrate that the random effects do indeed contain a substantial amount of spatial information which we can recover by extrapolation. However, there is also a component that cannot be explained spatially and is therefore lost in the extrapolation process (see Figures S15 and S16 in Text S1).

**Table 4:** Correlations (95% CIs from 1000 bootstrap samples) between the fitted and extrapolated random effects for models fitted to night and day time temperatures.

	night	day
Intercept	0.57 (0.43–0.68)	0.70 (0.57–0.80)
Night surface temperature	0.68 (0.59–0.77)	0.73 (0.62–0.83)
Day surface temperature	0.62 (0.46–0.77)	0.60 (0.46–0.74)
EVI	0.47 (0.26–0.63)	0.58 (0.41–0.71)
MIR	0.40 (0.22–0.56)	0.45 (0.30–0.59)
Rainfall	0.68 (0.57–0.78)	0.75 (0.61–0.85)

Fitted random effects are obtained from the full model, whereas extrapolated random effects are from the validation model extrapolated to the excluded 10% of locations.

**Table 5:** Variances of the random effects of the locations excluded from the 90% dataset for the model fitted to all locations, and the model fitted to 90% of locations, extrapolated to the remaining 10% of locations.

	Intercept		Night surface temperature		Day surface temperature		EVI		MIR		Rainfall	
	Night	day	night	day	night	day	night	day	night	day	night	day
full fit	17	19	0.57	0.45	0.41	0.44	1.3	0.58	1.9	2.4	0.04	0.056
extrapolation	6.5	11	0.22	0.27	0.14	0.22	0.36	0.27	0.42	0.81	0.021	0.036

We then proceeded to compare the temperature data and estimates from the full and validation model at the 10% of locations excluded from the validation dataset. We used the raw temperature time series aggregated to 64 time points per year (from the input data or as estimated by the models) and the temperature time series smoothed by Fourier transform (from the input data or estimated by the models), which describe the typical seasonal mean temperature. As the raw observed temperature time series is the “gold standard” that we aimed to predict with our model, we evaluated the differences between this and the estimated raw and smoothed time series, using the root mean squared differences to quantify the goodness of fit (Table 6). The fitted time series differed by typically around ±2°C from the raw observed time series – a substantial difference. When considering the differences between the smoothed time series and the raw observed temperature time series, it becomes clear that there is a lot of weekly variation around the seasonal mean. This weekly variation is not captured very well by the model fits, but the smoothed versions of the estimated temperatures from both the full and the validation model are very similar to the smoothed observed temperature time series, with in general less than 0.2°C difference between the two. For the extrapolated validation fit there was typically a less than 0.6°C difference for both day and night time temperatures.

**Table 6:** Root mean squared differences between the raw observed temperature time, series aggregated to 64 points per year, and the estimated time series from both the full fit and the validation fit, as well as smoothed versions of the raw data, full fit and validation fit.

	night		day	
	Raw	smoothed	raw	smoothed
observed	–	1.58	–	2.03
full fit	1.50	1.70	1.72	2.15
validation fit	2.02	2.15	2.21	2.54

Results are shown only for the 10% of locations excluded from the validation dataset.

A lower root mean squared difference indicates a better fit.

### Malaria Transmission

**Figure 4:**
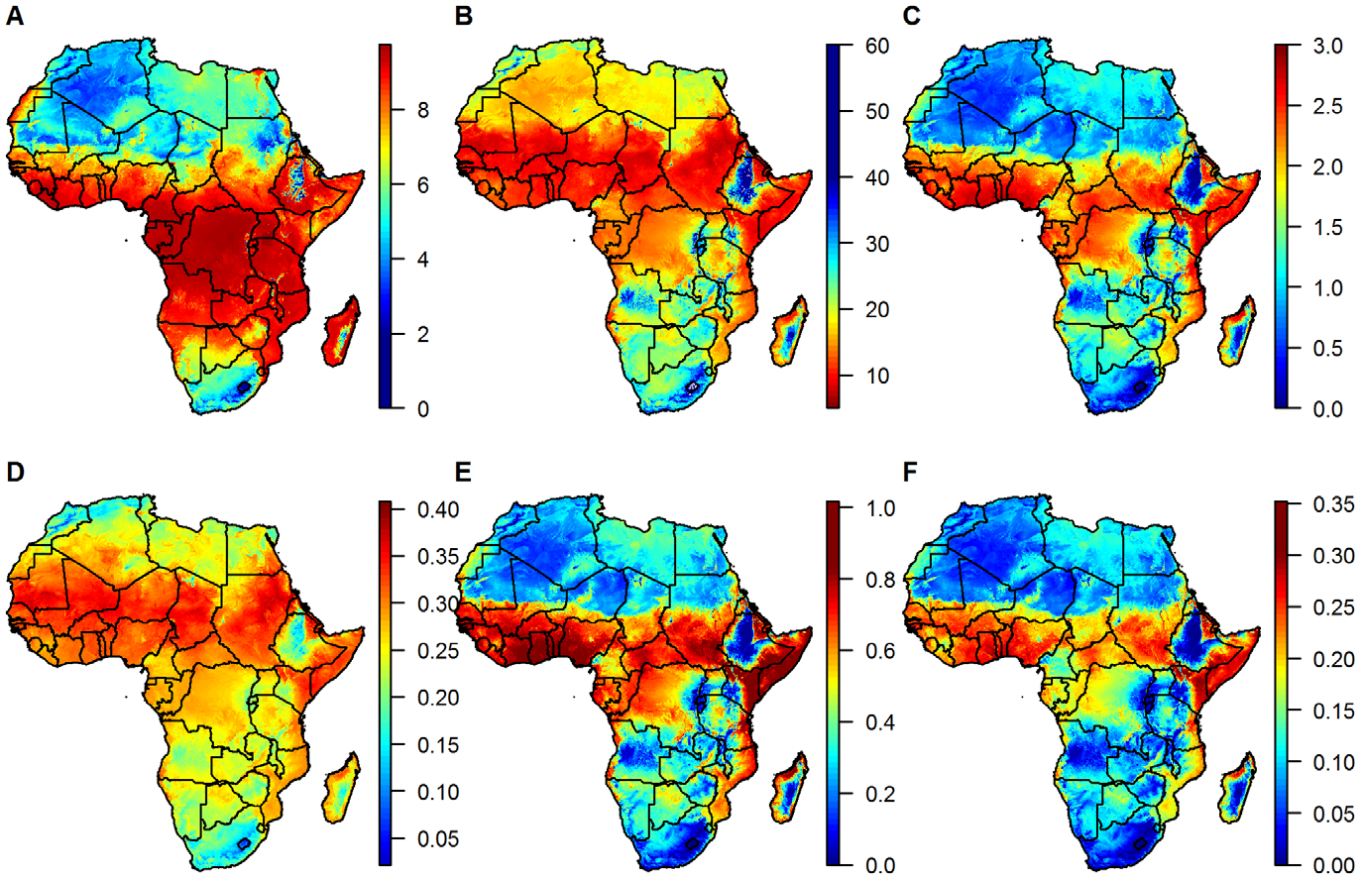
Maps of the mean annual malaria transmission parameters. (A) average mosquito life span, (B) mean extrinsic incubation period (cut off at 60 days), (C) mean extrinsic infectious period, (D) mean biting rate, (E) mean number of infectious bites per infected mosquito and (F) temperature suitability index.

The estimated annual mean mosquito life span ranges from close to zero in some highland regions to around nine days, with durations of over a week in most of sub-Saharan Africa (Figure 4A). The annual mean of the extrinsic incubation period for *Plasmodium falciparum* ranges from around 6 days in the Sahel and sub-Sahel regions, through 20 days in parts of Northern and Southern Africa, to 60 days or more in highland areas (Figure 4B). Combining the extrinsic incubation period with temperature dependency in mosquito survival, we estimated the longest infectious periods (and shortest incubation periods) are to be expected in West, Central and Eastern Africa (Figure 4C), coincident with areas known to support high malaria transmission intensity. The average biting rate (Figure 4D) echoes the patterns of temperature with the highest biting rates in the Sahara desert (ignoring other factors that determine mosquito abundance). Combining this with the duration of the infectious period we get a concentration of the number of infectious bites per infected mosquito in the belt between 5° and 15° latitude as well as further south on the East coast of the continent, and even more focus in West and East Africa for the temperature suitability index (Figure 4E and F).

**Figure 5:**
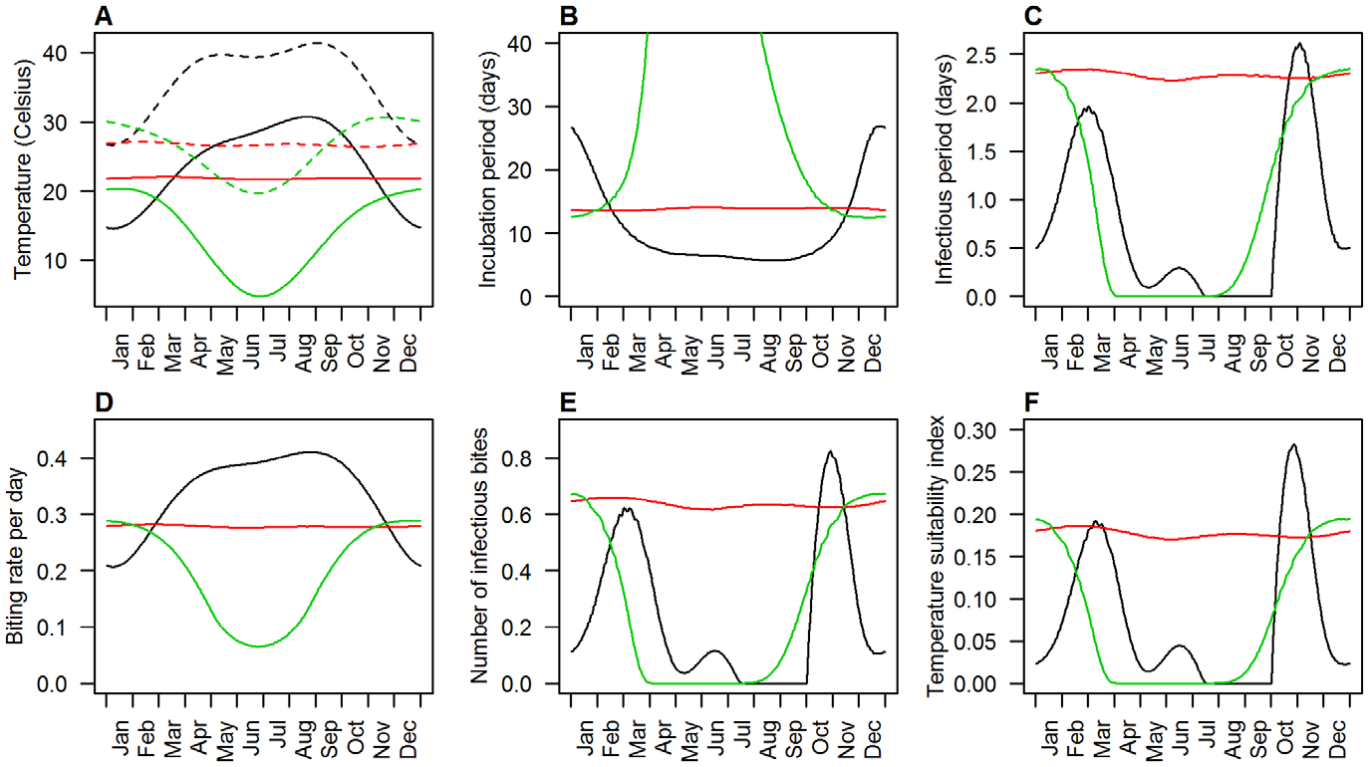
Seasonality of temperatures as well as several malaria transmission parameters for three selected locations. (A) Daily minimum (solid lines) and maximum (dashed lines) air temperatures, (B) extrinsic incubation period, (C) extrinsic infectious period, (D) daily biting rate, (E) average number of infectious bites per infected mosquito and (F) temperature suitability index. Locations as marked in Figure 1: Black, red and green lines for L1, L2 and L3, respectively.

The seasonal variation of incubation and infectious periods show the complex interplay of the temperature dependencies (see Figure 5 for three selected locations or Movie S1). Short infectious periods (and therefore lower transmission intensities) are observed whenever the incubation periods are long, due to the low probability that the mosquito survives to become infectious. However, short incubation periods do not always lead to long infectious periods, as in areas where more extreme temperature fluctuations are observed diurnally and seasonally there is a competing risk that mosquitoes will die more quickly. When taking the biting rates into account to calculate the number of infectious bites and the temperature suitability index, the overall seasonal patterns remain similar to that of the infectious period, but are modulated somewhat between locations.

To demonstrate the importance of using the correct type of temperature data we evaluated the malaria transmission indices using land surface temperatures as input data, showing that the results are strikingly different in magnitude as well as in seasonal patterns (see Files S1 and S2). This difference is mainly caused by the reduced mosquito life span due to the very high day-time land surface temperatures in many areas, and means that the area of suitability would be predicted to be much more concentrated on tropical regions in Central and West Africa when using land surface temperatures.

### Dataset Publication

The Fourier transforms of the estimated air temperatures are published as Datasets S1 and S2 for night and day time temperatures, respectively, at a resolution of 0.1° longitude and latitude; for information on recovering time series from the Fourier transforms see Text S1 and the worked examples in R and Excel in Calculations S1 and S2, respectively. For higher resolution datasets at 30″ = 1/120° or datasets of the ecological covariates used in the model fitting please contact the authors.

## Discussion

Previous studies have demonstrated the importance of temperature on the developmental stages of Anopheles species [7], [8], [54] which in turn partly determine the suitability of localities for malaria transmission [20]. Whilst differences between land-surface, air and indoor temperature have been recognised [29], [59], the absence of high resolution (both spatially and temporally) data on air surface temperature has limited translation of these results to maps of areas of malaria transmission suitability. Our results demonstrate that air and surface temperatures across Africa differ substantially, as demonstrated previously for specific locations [59]. We find that air temperatures can be predicted with a high degree of accuracy at a continental scale using surface temperatures and other climatic and ecological covariates, consistent with previous studies that identify relationships between vegetation, land cover and air temperature [60], [61], [62], [63].

Although temperature and other climatic variables fluctuate substantially on a daily or weekly basis in addition to the more regular diurnal fluctuations, the average seasonal pattern is often more relevant to understand the impact of interventions against malaria. These can be obtained by smoothing time series through Fourier transforms, and reconstructing them by only including the modes of interest. Our results demonstrate that the mean and the annual and biannual modes give a good representation of seasonality across Africa while smoothing out the high frequency fluctuations. The use of Fourier transforms to represent the time series also means that the size of the dataset can be reduced substantially, as there are only 5 parameters necessary for each location to describe the mean and first two modes, as opposed to a much larger number of time points if the raw time series are used. This becomes important when large spatial areas are to be represented at high spatial resolution.

The validation of our model fit revealed substantial deviations from the raw temperature time series. This is primarily due to weekly fluctuations that are not captured well. However, when comparing the smoothed time series derived through Fourier Transforms, these differences are substantially reduced, showing that our model captures the overall seasonal patterns well. It is important to keep this in mind and only use datasets such as ours when the random weekly fluctuations are not relevant, for instance if the typical pattern across several years is of interest, in which case the short-term random fluctuations would be a nuisance rather than useful information.

To assess the impact of temperatures on malaria transmission we focused on the adult mosquito stages of the transmission cycle, investigating the effect of air temperatures on mosquito survival, biting rate and parasite development inside the mosquito [6], [64], using a similar approach to that used in other work [20]. However, our definition of the temperature suitability index differs from that study in that we have not taken into account the effect that mortality temperature dependence has on mosquito abundance. This is because we felt that mosquito abundance is much more dominated by factors such as rainfall patterns. However, the recruitment rate of adult mosquito emergence will also depend on temperatures, albeit water rather than air temperatures. Here we have focussed on the substantial temperature dependence of the biting rate, which causes a concentration of the temperature suitability in the Sahel and sub-Sahel belt as there the trade-off between mortality and biting rates, which both increase with increasing temperature, appears to be optimal.

The areas that we identified with a high average temperature suitability index, indicating the potential for malaria transmission based on temperature, broadly encompass the areas in which malaria is endemic [65]. However, temperature suitability is only one environmental factor that determines the potential for malaria transmission and thus some areas that are identified here do not in reality support high levels of malaria. In particular, temperature alone identifies parts of the Horn of Africa (Southern Sudan, Somalia, Northern Kenya and parts of Ethiopia) as having higher potential for transmission than is truly the case. Furthermore other climatic factors influence Anopheles population dynamics, including the dependence of larval development dependent on water temperatures and vector abundance on the availability of suitable habitats which is related to rainfall patterns [4], [5].

We used the modelled air temperature time series fitted to approximately weekly temperature data smoothed by Fourier transforms to evaluate the impact of temperature on the malaria transmission potential. We also incorporated diurnal fluctuations which are important for malaria transmission dynamics [31], using hourly timesteps in the calculations of the malaria transmission indices in which we interpolated between the smoothed day and night time temperatures. While our approach in determining malaria transmission intensity is similar to that used in [20], there long-term monthly mean, minimum and maximum temperatures were used, which were interpolated to obtain smooth temperature curves. In contrast, our approach is more likely to give a realistic picture of the actual seasonality due to the richer temperature data used as input. A further difference is the period of input data. Whilst [20] used data from 1950 to 2000, which would be appropriate to explain historical patterns of malaria, our datasets cover the period from 2003 to 2006, making our study well-suited to explain currently observed patterns of malaria transmission, and for use in projections into the future.

In summary, we have estimated air temperatures at spatial resolution of up to 30 arcseconds longitude and latitude (approx. 1 km) and published these datasets for use in further research. We then used the synthetic datasets we generated to highlight the areas across Africa where temperature profiles are favourable to malaria transmission. Combined with other climate-dependent factors influencing malaria transmission intensity, such as the abundance of competent vector species, these data can give insights into geographic variation in malarial transmission intensity patterns of seasonality that can help to inform malaria control and are crucial for developing more sustainable vector control approaches such as integrated vector management that are urgently needed in the face of increasing resistance of mosquitos to conventional insecticides [66].

## Supporting Information

Text S1 **Further details of the Fourier transforms and the model validation and extrapolation.**
(PDF)

Movie S1 **Movie of the extrinsic incubation and infectious periods, the biting rate and the temperature suitability index throughout the year across Africa.**
(GIF)

File S1 **Maps of the mean annual malaria transmission parameters, calculated using land surface instead of air temperatures as input data.** Legend as in Figure 4.
(TIF)

File S2 **Seasonality of land surface temperatures as well as several malaria transmission parameters evaluated using land surface instead of air temperatures as input data.** Legend as in Figure 5.
(TIF)

Dataset S1 **Fourier transforms of the modelled night time air temperatures across Africa at a spatial resolution of 0.1° longitude and latitude.**
(ZIP)

Dataset S2 **Fourier transforms of the modelled day time air temperatures across Africa at a spatial resolution of 0.1° longitude and latitude.**
(ZIP)

Calculation S1 **Example demonstrating how to calculate time series from the Fourier transforms published in Datasets S1 and S2 as an R script.**
(R) Click here for additional data file.

Calculation S2 **Example demonstrating how to calculate time series from the Fourier transforms published in Datasets S1 and S2 as an Excel spreadsheet.**
(XLS)
