# Supplementary material for: Estimating Air Temperature and Its Influence on Malaria Transmission across Africa
Source: PLoS One. 2013 Feb 20;8(2):e56487. doi: 10.1371/journal.pone.0056487 (PMC3577915; doi:10.1371/journal.pone.0056487)
Supplement: Text S1 — Further details of the Fourier transforms and the model validation and extrapolation. (PDF) [file pone.0056487.s001.pdf]

Estimating of air temperature and its influence on malaria transmission across Africa
Supplementary Text S1

Tini Garske*, Neil M Ferguson, Azra C Ghani

MRC Centre for Outbreak Analysis and Modelling, Department of Infectious Disease Epidemiology, Imperial College London, UK

*Corresponding author: t.garske@imperial.ac.uk

Contents

Fourier transforms 2

Identifying important modes 3

Reconstructing smoothed time series from the Fourier Transforms 4

Missing data 8

Sensitivity analysis of the ring radius 10

Model validation and extrapolation 12

References 17

Fourier transforms

The Fast Fourier transform[1] employed in this study requires input data at $N = 2^i$ equally spaced time points $t_k = k\Delta$, where Δ is the time step and $k = 0,1,2,\ldots,N-1$, and in turn calculates the Fourier transform for 2^i equally spaced frequencies, $f_\ell = \frac{\ell}{N\Delta}$, $\ell = 0,1,2,\ldots,N-1$. As we expect the annual frequencies to be important, we use data from 2^m years with 2^j data points per year (thus $\Delta = 2^{-j}\,\text{years}$) to ensure that the annual frequencies are indeed computed, and re-label the frequencies by $n = 2^{-m}\ell$, such that $f_{n=1}$ and $f_{n=2}$ always refer to the annual and biannual frequencies etc, irrespective of the length of the input time series. Here, $2^m = 4$ and $2^j = 64$, yielding 256 datapoints in each time series with a time step $\Delta = 5.707\,\text{days}$

The Fourier Transform of the time series $h_k = h(t_k)$ is given by

$$H_n = \sum_{k=0}^{N-1} h_k e^{\frac{2\pi ikn}{2^{-m}N}},$$

whereas the reverse transform is

$$h_k = \frac{1}{N} \sum_{n=0,2^{-m},\ldots}^{2^{-m}(N-1)} H_n e^{\frac{-2\pi ikn}{2^{-m}N}}.$$

In our case, the time series h_k is real, and therefore the Fourier coefficients follow $H_{2^{-m}N-n} = H_n^*$, with H_0 and $H_{2^{-(m+1)}N}$ real. In this case, the exact time series can be reconstructed from the Fourier coefficients as

$$h_k = \frac{1}{N}H_0 + \frac{2}{N}\sum_{n=2^{-m}}^{2^{-m}(N/2-1)}\left[\operatorname{Re}(H_n)\cos\left(\frac{2\pi kn}{2^{-m}N}\right) + \operatorname{Im}(H_n)\sin\left(\frac{2\pi kn}{2^{-m}N}\right)\right] + \frac{(-1)^k}{N}H_{N/2}. \quad \text{(S1)}$$

An approximation of the time series based on the most important modes only can be obtained by setting $\operatorname{Re}(H_n) = \operatorname{Im}(H_n) = 0$ for all modes n to be neglected. The value at an arbitrary time t can be calculated by letting $k = t/\Delta$ vary continuously. Worked examples of reconstructing the time series from the Fourier transforms dataset published alongside this paper are given in the form of an R script and an Excel worksheet in Supplementary Calculations S1 and S2, respectively.

Identifying important modes

The contribution or weight w_n of each mode (determined by its frequency $f_n = \frac{n}{N\Delta}$) to the overall time series can be quantified by calculating the relative amplitude of the oscillation,

$$w_n = \frac{\mathrm{Re}^2(H_n) + \mathrm{Im}^2(H_n)}{\sum_{l=0}^{N-1}\left[\mathrm{Re}^2(H_l) + \mathrm{Im}^2(H_l)\right]}.$$

We averaged the weight of each mode over the 1113 locations across Africa on the 1.5° grid for the time series of all time series variables used in this study (Figure S1) and found that generally slower frequencies are more important than fast fluctuations, and by far the most important mode is indeed the annual mode, followed by the bi-annual mode for all datasets apart from the EVI and MIR time series, where even slower frequencies take the second and third ranks. These slower than annual frequencies determine fluctuations between years and are therefore not of interest if results should be generalised to years outside the range of the input time series. Overall, frequencies that are integer multiples of the annual frequency tend to be more important than other frequencies of similar magnitude, particularly in the low-frequency regime of up to around 5 oscillations per year, reflecting the fact that the seasonality is driven by annual cycles.

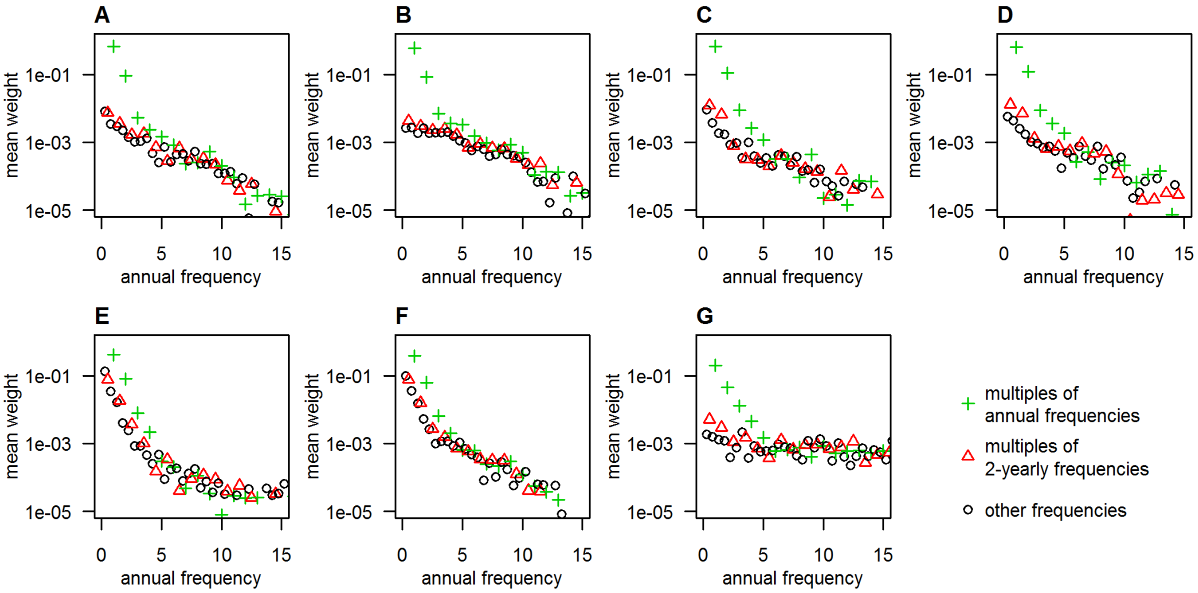

Figure S1: Importance of various frequencies in the Fourier Transforms. Average weight of each frequency across Africa is plotted against the frequency for night time air (A) and surface (B) temperatures, day time air (C) and surface (D) temperatures, EVI (E), MIR (F) and rainfall (G).

Reconstructing smoothed time series from the Fourier Transforms

We reconstructed the time series from the Fourier transforms, but only including the terms of the most important modes, starting with the constant term H_0 only, which yields values varying across locations, but constant throughout the year, then including the constant term and the annual mode which results in sinusoidal oscillations around the mean value, and finally adding successively higher modes of integer multiples of the annual frequency. We calculated correlations between the time series aggregated to 64 points per year, prior to Fourier transforms, and the Fourier reconstructions of these time series evaluated at the same time points (Figure S2). The correlations with the constant term only vary considerably between the datasets and are surprisingly high for some datasets indicating a low level of seasonal variation compared to the spatial variation. The correlations steeply increase with inclusion of the first two annual modes, but then reach a plateau, and therefore including further frequencies in the reconstruction does not improve the capture of the general seasonal patterns seen.

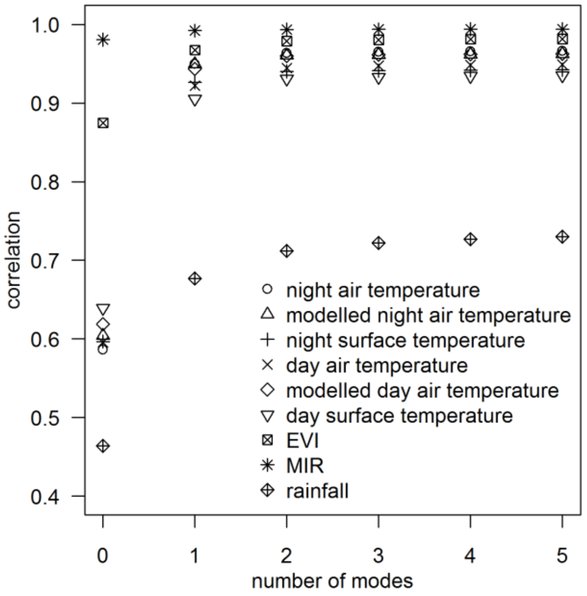

Figure S2: Correlations between the original and reconstructed time series for the different time series datasets. Original time series aggregated to 64 points per year vs. the reconstruction of the time series from the Fourier transform, including the constant term and up to 5 modes (the first 5 integer multiples of the annual frequency).

Plots of the original time series compared to the Fourier reconstructions based on the first few annual modes show that the annual mode captures the majority of the seasonal oscillations, whereas the biannual mode serves to hone the shape to follow the observed patterns more closely, and in most places the triannual mode does not contribute much to the overall visual match (Figure S3 to Figure S5), confirming the results found in comparing the correlations. We therefore use the reconstructed time series based on the constant term H_0 and the annual and biannual modes, H_1 and H_2, respectively, to describe the overall seasonality. This reduces the number of parameters to be stored from 256 to 5 per location. The observed values vary around the smoothed curves with a standard deviation of the difference of 1.56°C, 1.57°C and 2.04°C for air, modelled air and surface night temperatures, 2.03°C, 1.66°C and 3.40°C for air, modelled air and surface day temperatures, 0.030 and 0.023 for EVI and MIR and 2.13 mm per day for rainfall.

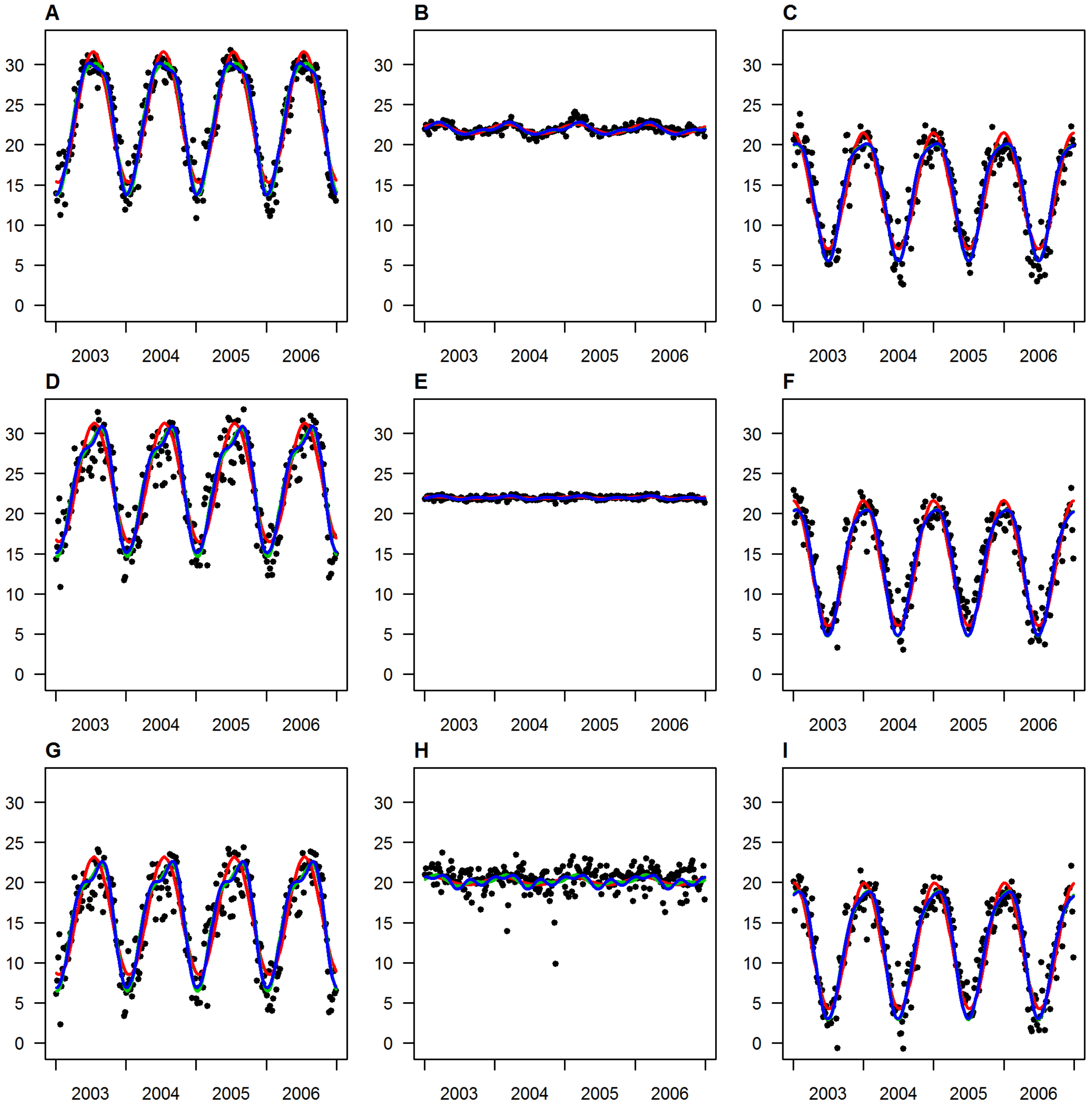

Figure S3: Night time temperatures for three selected locations. Top (panels A, B, C) for the air temperature, middle (D, E, F) for modelled air temperatures and bottom (G, H, I) for surface temperatures for the locations marked in Figure 1 of the main text: Latitudes 19.5, 0 and -25.5 and longitudes 0, 19.5 and 25.5 for left (panels A, D, G), middle (panels B, E, H) and right (panels C, F, I), respectively. Black circles: Time series aggregated to 64 points per year, lines: time series reconstructed from the Fourier transform, including the annual (red), the annual and biannual (green) and the annual, biannual and triannual (blue) modes.

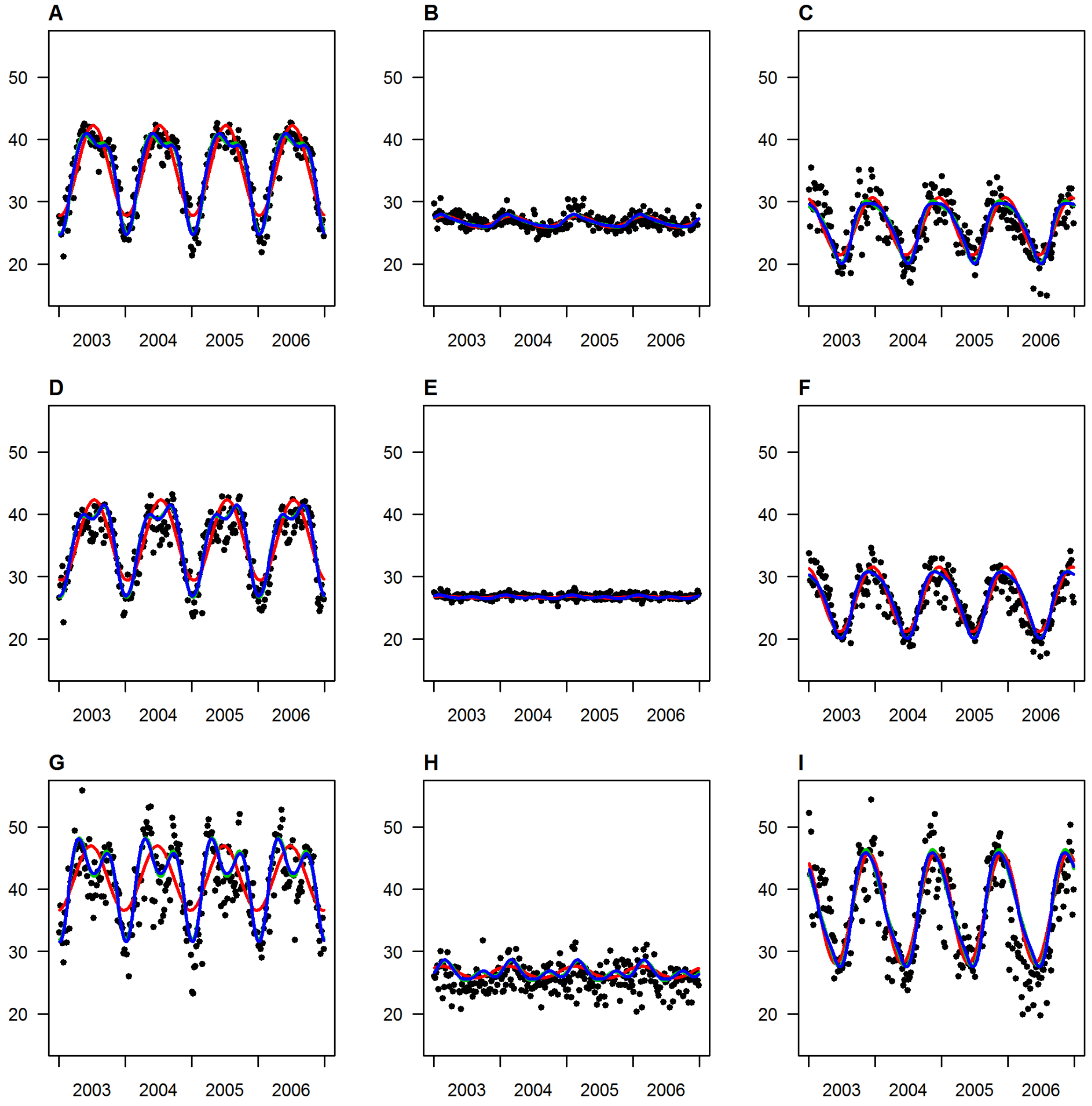

Figure S4: Day time temperatures for three selected locations. Legend as for Figure S3, but for day time temperatures.

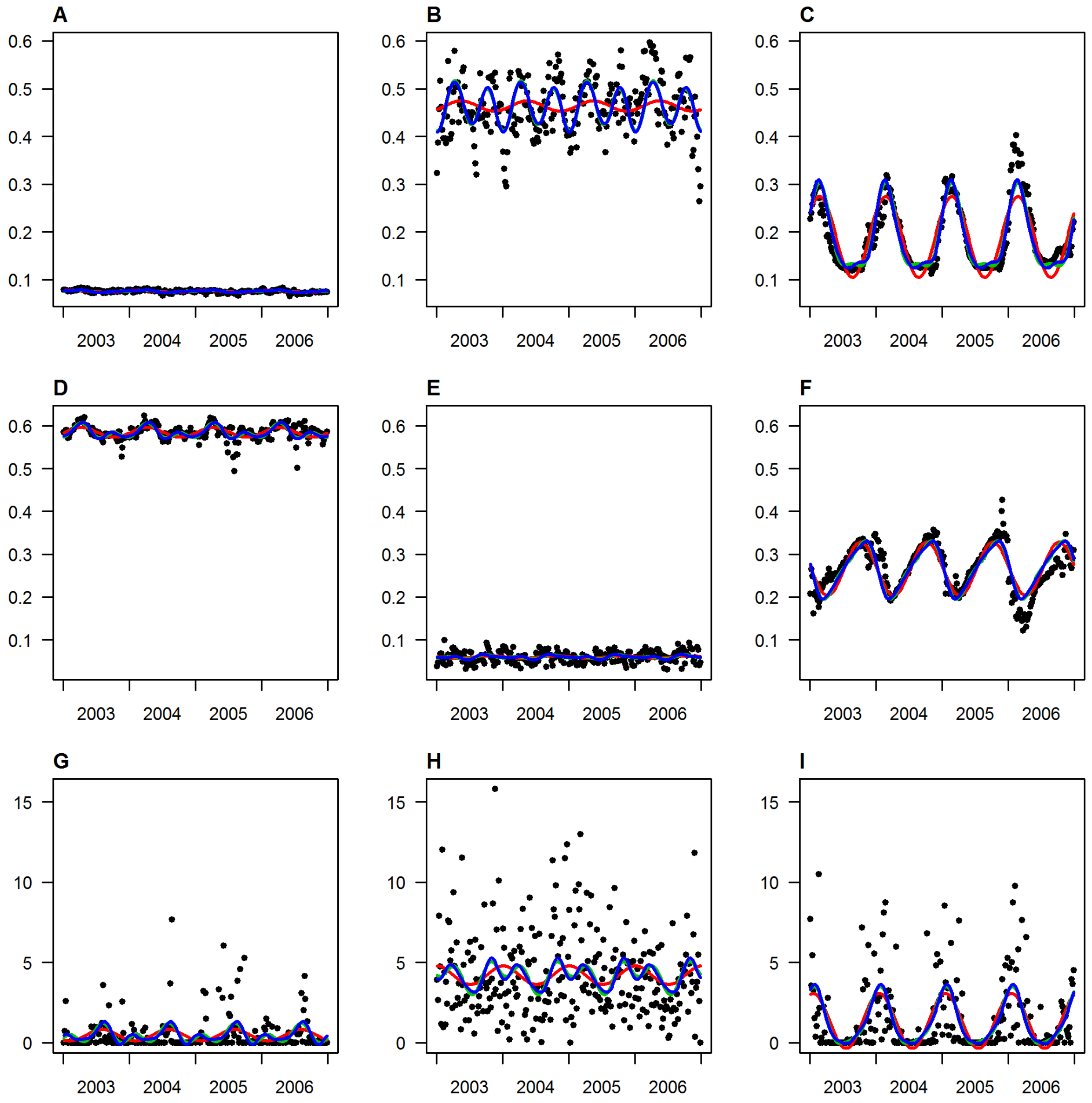

Figure S5: EVI, MIR and rainfall for three selected locations. Legend as for Figure S3, but for EVI (top, panels A, B, C), MIR (middle, panels D, E, F) and rainfall (bottom, panels G, H, I).

Missing data

Some of the time series datasets contain a substantial amount of missing data (due to reasons such as cloud cover). In order to analyse the proportion of missing data we restricted our analysis to a grid of 1.5° resolution, see Figure S6. The largest amount of missing data is found for the night time temperatures, with over 30% of data points missing in some locations. For the day time surface temperatures, in most locations we had complete time series, but a few locations showed up to 17% of missing data. The EVI and MIR datasets shared the missing data patterns. For these, locations had between 0 and 4 of the 184 data points missing (up to 2.2%), with more missing data in the north than the south. In the rainfall dataset, only two locations of the 1113 locations on the grid had one data point missing, all other locations had complete daily time series.

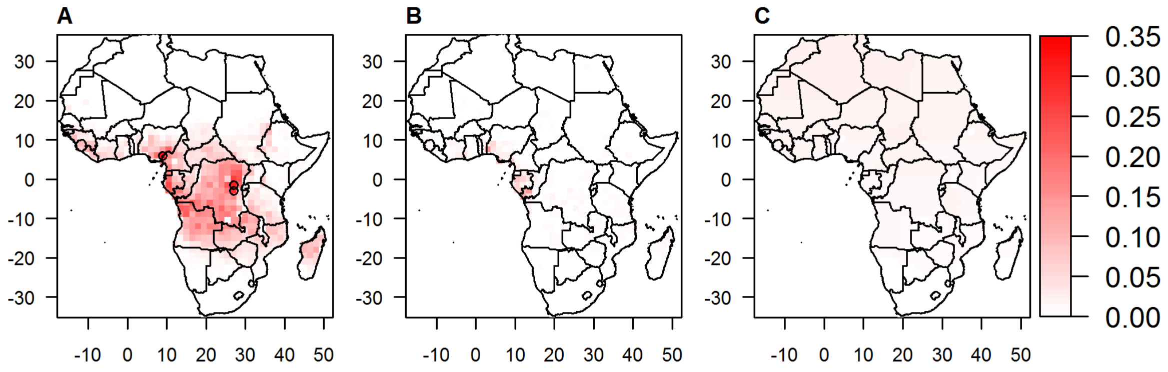

Figure S6: Proportion of missing data. (A) Night time surface temperature, (B) day time surface temperature and (C) EVI and MIR. The three locations with the highest proportion of missing data for night time temperatures are highlighted by black circles in panel A.

For some locations we found lengthy periods without any night or day time temperature measurements, which we interpolated linearly between the neighbouring data points. This could potentially affect the Fourier transforms and render the reverse transformations unreliable. However, the locations with the most missing data were in areas where the annual variation in temperature was low (Figure S7), and the time series reconstructed from the Fourier transforms appeared to be reasonable approximations of the seasonal patterns in the original measurements even for the locations with the largest proportion of data missing (Figure S8).

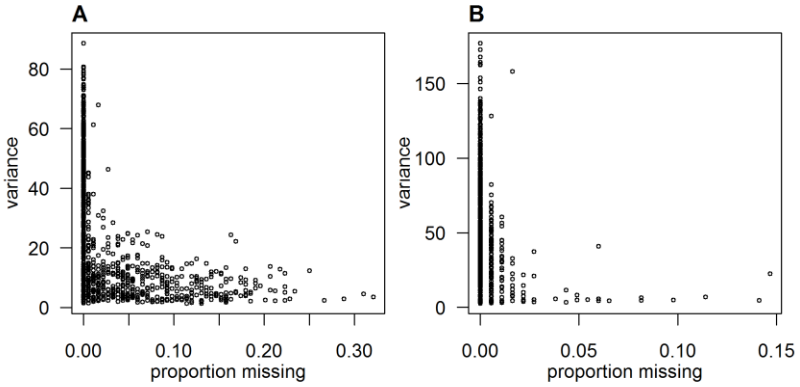

Figure S7: Variance of the surface temperature time series vs the proportion of time points missing by location. (A) night time and (B) day time.

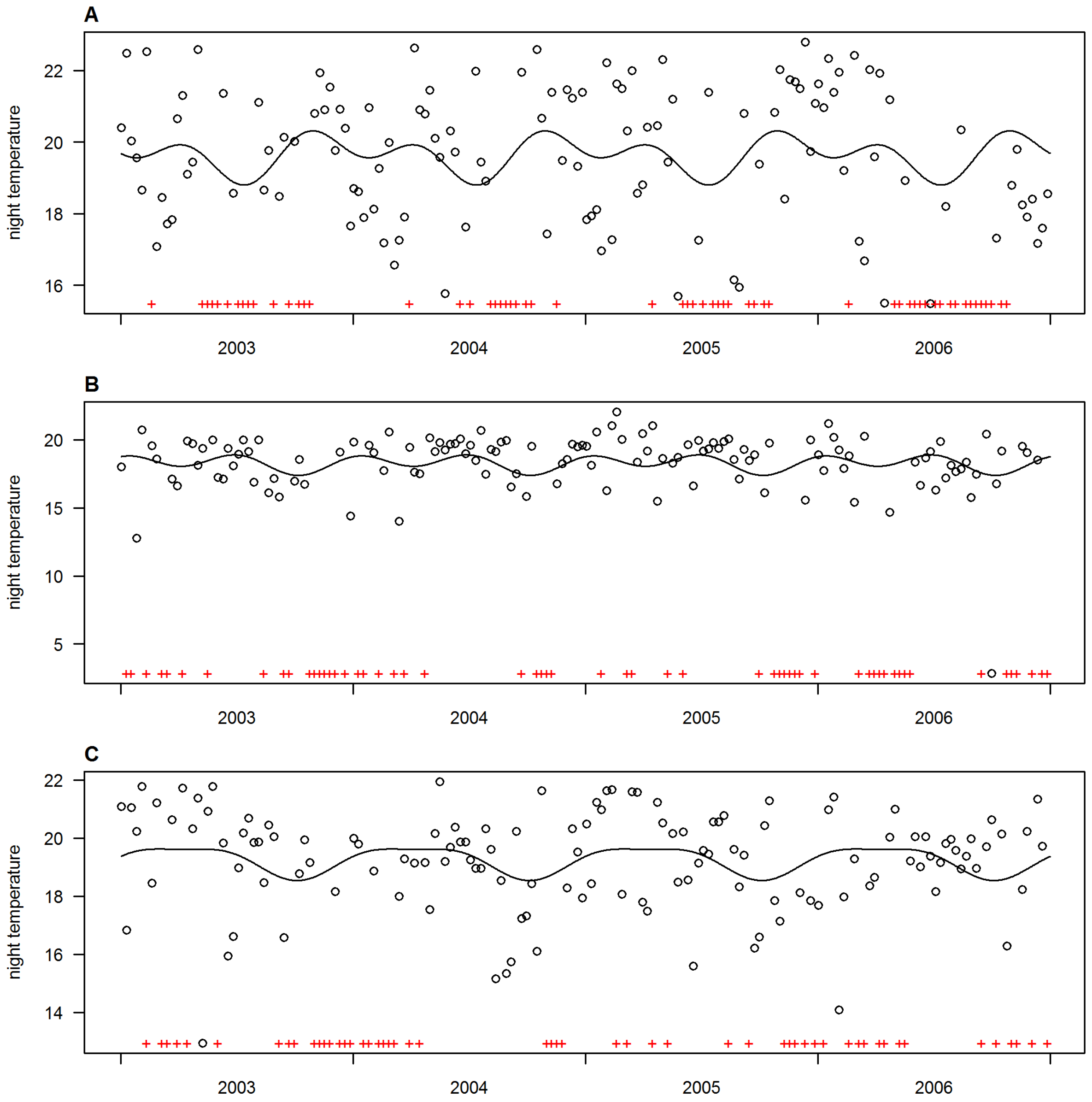

Figure S8: Night surface temperature time series of the locations with the highest proportion of missing data. Locations as marked in Figure S6A: longitude 9°, 27° and 27° and latitude 6°, -1.5°, -3° for A, B and C, respectively. Black circles are the actual data points, red crosses mark the missing time points, and black lines show the reconstruction via Fourier transforms.

Sensitivity analysis of the ring radius

We aggregated the environmental data within a radius of 5km in order to represent the surrounding area on a length scale relevant for local malaria transmission dynamics rather than use data from single point locations. As the radius of 5km was chosen fairly arbitrarily, we investigated the effect of different radii on the datasets, using the night and day time surface temperatures as examples. Results for the other datasets were very similar.

Firstly, the spatial aggregation within a ring meant that there were more potential input datapoints the larger the ring radius, and therefore the proportion of data missing was smaller for larger radii (Figure S9). While datasets with smaller ring radius contained slightly more extreme values, the differences between the datasets were small, with all pairwise correlations between the 2km, 5km and 10km ring radius datasets above 0.98 for both night and day surface temperatures (Figure S10).

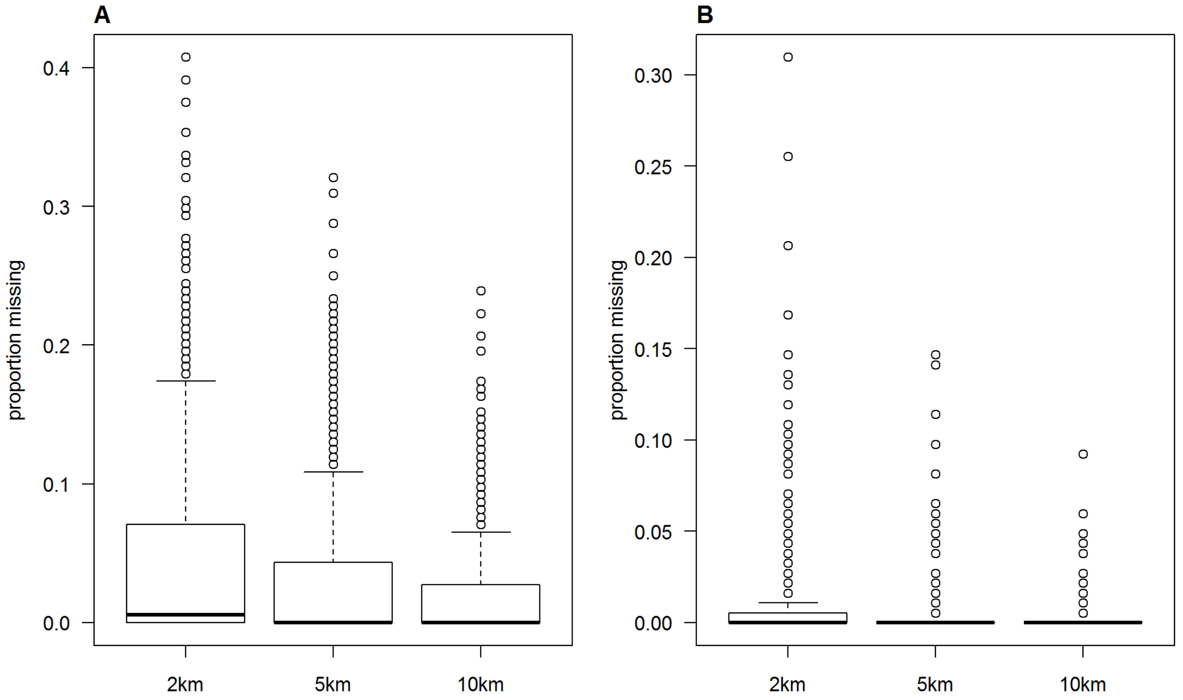

Figure S9: Proportion of data points missing for various ring sizes for night and day time temperatures (A and B, respectively). Boxplots show the median (thick horizontal line), interquartile range (box), continuous range (whiskers) and outliers (circles).

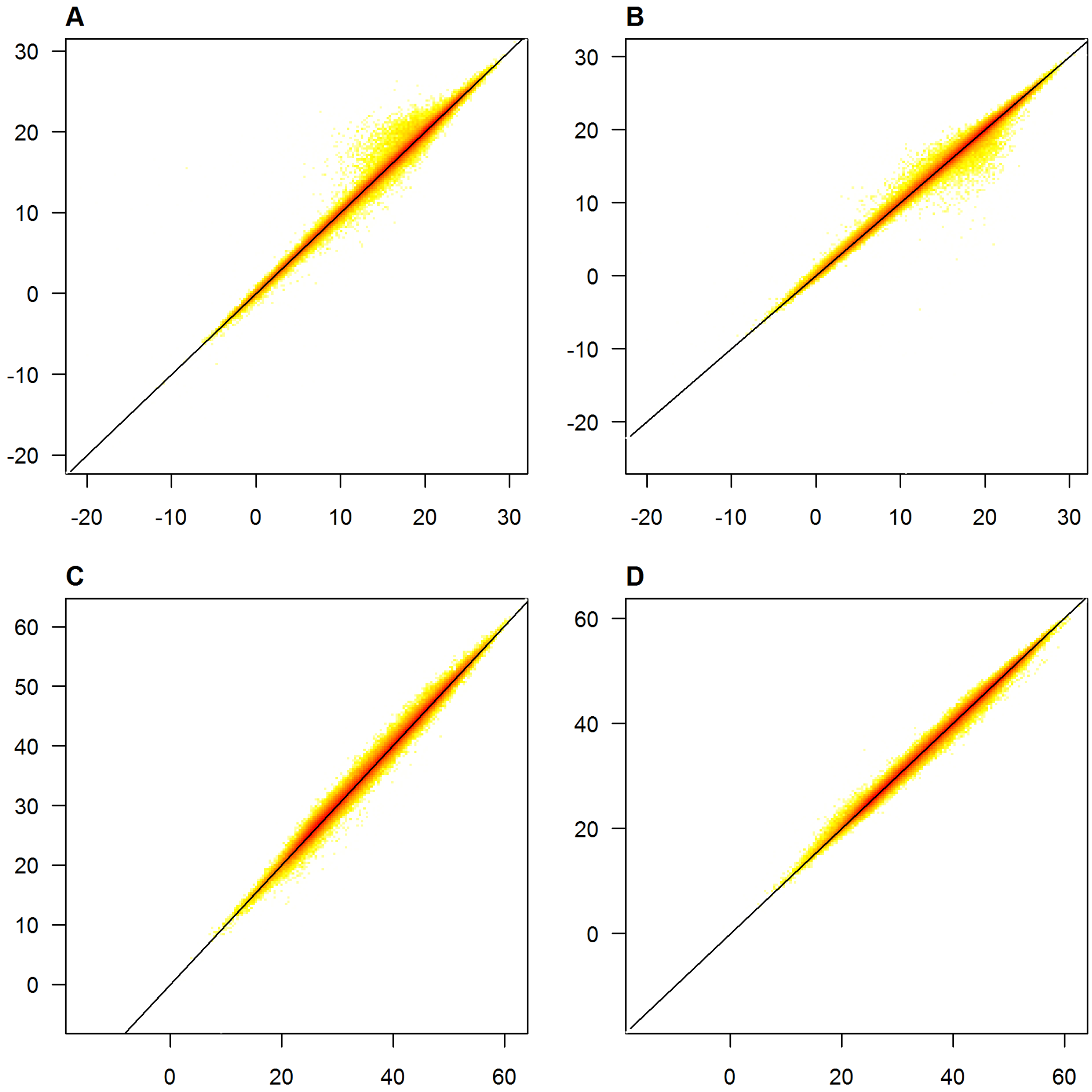

Figure S10: Density of the surface temperatures for a 2km radius (panels A and C) and a 10km radius (panels B and D) against the surface temperatures for a 5km radius for night (A and B) and day time temperatures (C and D). Temperatures are shown in Celsius, density on a logarithmic scale. Black lines indicate the identity.

Model validation and extrapolation

The approach of extrapolating the random effects to locations the model was not fitted to via ordinary kriging[2] only makes sense if there are spatial correlations between the random effects. Analysis of the random effects included in the model revealed considerable spatial correlations as described by the variograms shown in Figure S11 and Figure S12. We fitted functional forms of the exponential type to the observed variograms; the values of the fitted variogram parameters are given in Table S1, alongside the parameter values of variograms fitted to the random effects obtained from the validation model fit (fitted to 90% of the data), which are unsurprisingly rather similar.

For model validation, we used the random effects from the validation model to estimate the random effects at the locations excluded in the validation model, and compared these to the random effects obtained for these locations in the full model fit. The correlations between these were positive throughout, but somewhat variable in magnitude (Figure S13 and Figure S14), indicating that there was some spatial component to the random effects which could be captured in the extrapolation, but also some additional unexplained variation.

We then extrapolated the random effects from the full model fit using the fitted variograms. The random effects surfaces (Figure S15 and Figure S16) show that the extrapolated random effects had a lower variation than the random effects at the locations of the model fit, as the spatial correlations explained only part of the overall pattern of random effects; the remainder of the information inherent in the random effects was lost in the extrapolation.

Table S1: Parameter values of the variograms fitted to the random effects for night and day time air temperatures. Random effects obtained from the full fit and the validation model fitted to 90% of the data.

		Nugget		Sill		Range	
		full fit	validation	full fit	validation	full fit	validation
Intercept	night	6.74	6	10.3	11.1	3.78	3.5
	day	4.49	3.9	15.6	16.5	4.24	4
Night surface temperature	night	0.176	0.19	0.416	0.41	5.01	5.4
	day	0.069	0.061	0.418	0.43	3.79	3.7
Day surface temperature	night	0.063	0.059	0.289	0.29	3.15	3.1
	day	0.075	0.052	0.377	0.39	4	3.7
EVI	night	0.496	0.54	1.02	1.1	2.2	2.4
	day	0.348	0.35	0.411	0.5	4.66	4.3
MIR	night	1.17	1.2	0.822	0.85	3.99	4.5
	day	1.01	0.95	1.33	1.4	4.26	4.3
Rainfall	night	0.014	0.014	0.032	0.034	2.9	2.6
	day	0.0094	0.01	0.06	0.061	3.82	3.7

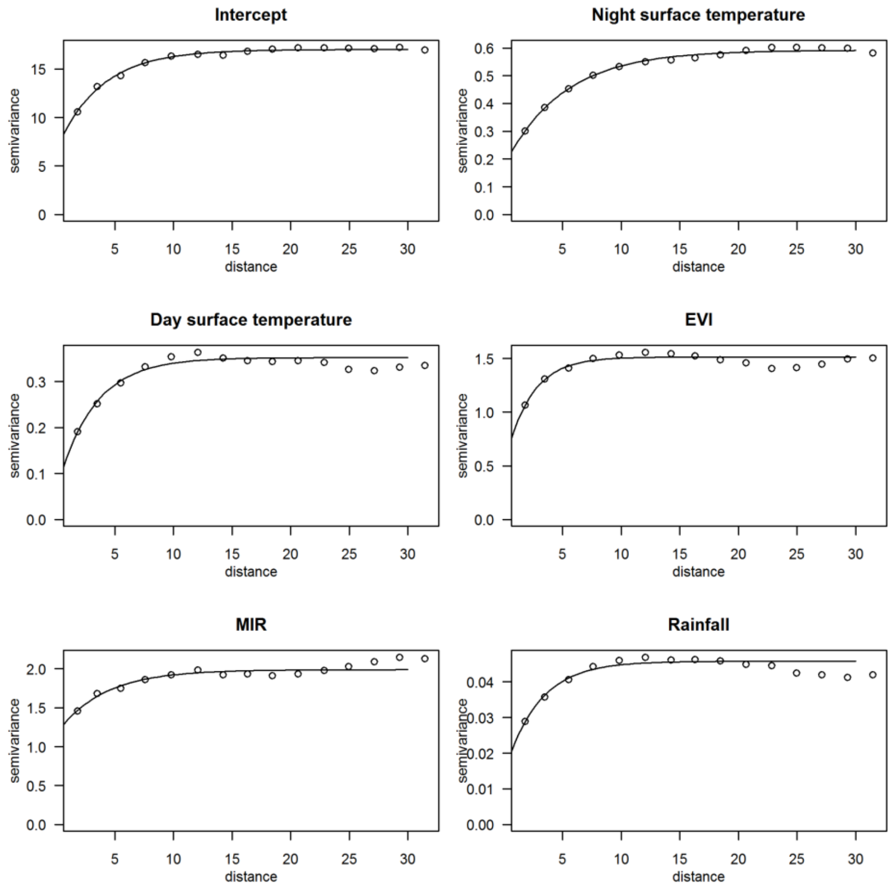

Figure S11: Observed (circles) and fitted (lines) variograms of the random effects from the full model fit fitted to night time air temperatures.

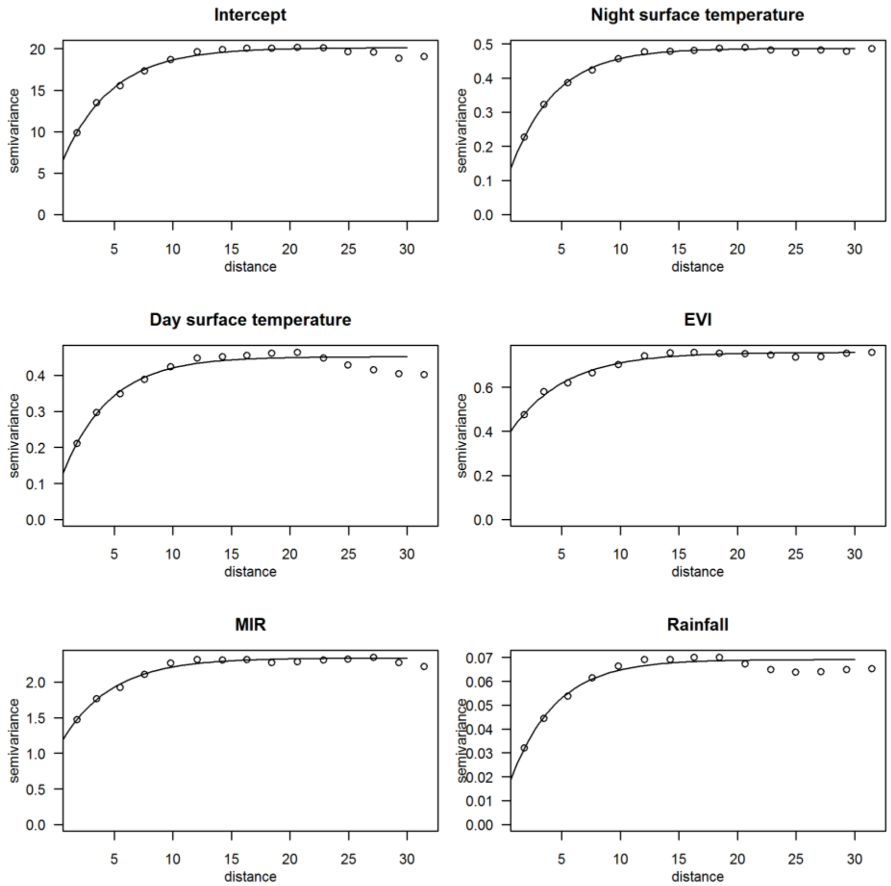

Figure S12: As Figure S11, but for day time air temperatures.

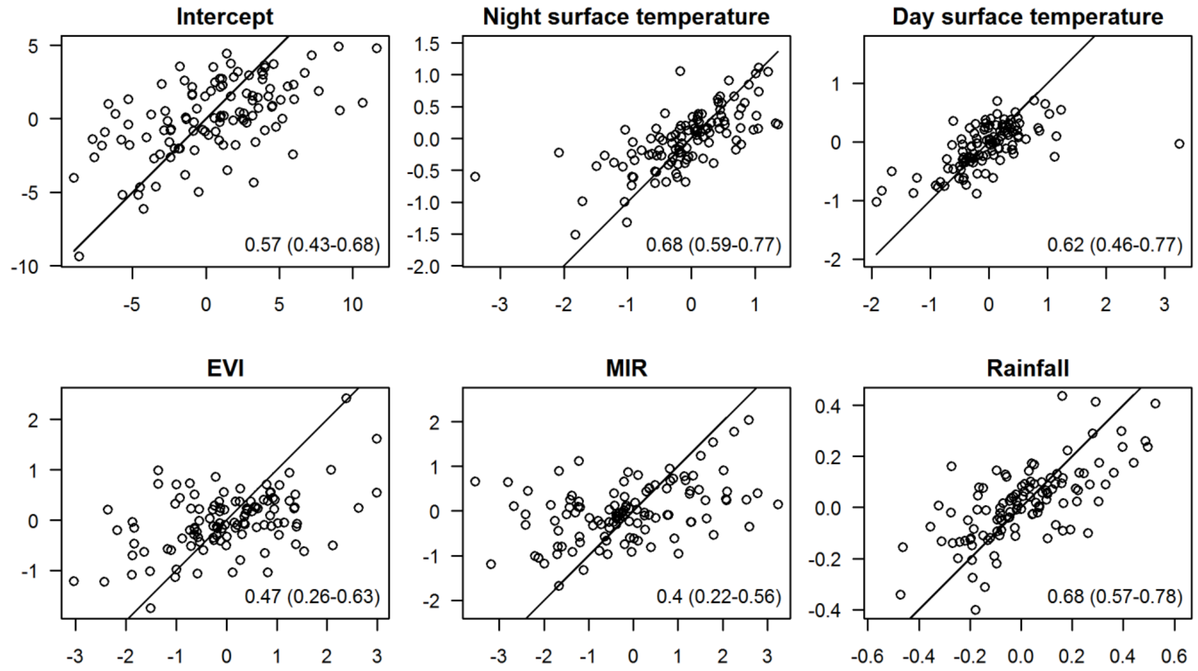

Figure S13: Comparison of the fitted and extrapolated random effects for night time temperatures. Random effects extrapolated to the excluded 10% of locations (vertical axis) vs random effects of the model fit to the full dataset (horizontal axis). Black line indicates unity. Correlations (95% CIs from 1000 bootstrap samples) are shown in the bottom right corner of each plot.

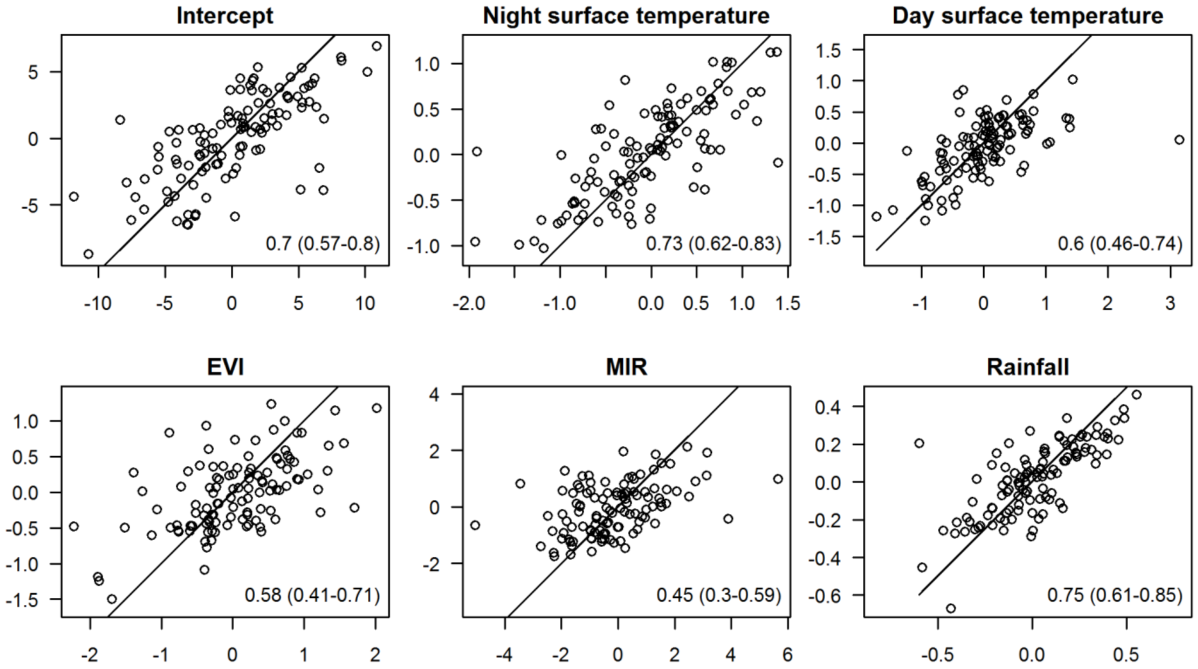

Figure S14: Comparison of the fitted and extrapolated random effects for day time temperatures. Legend as for Figure S13.

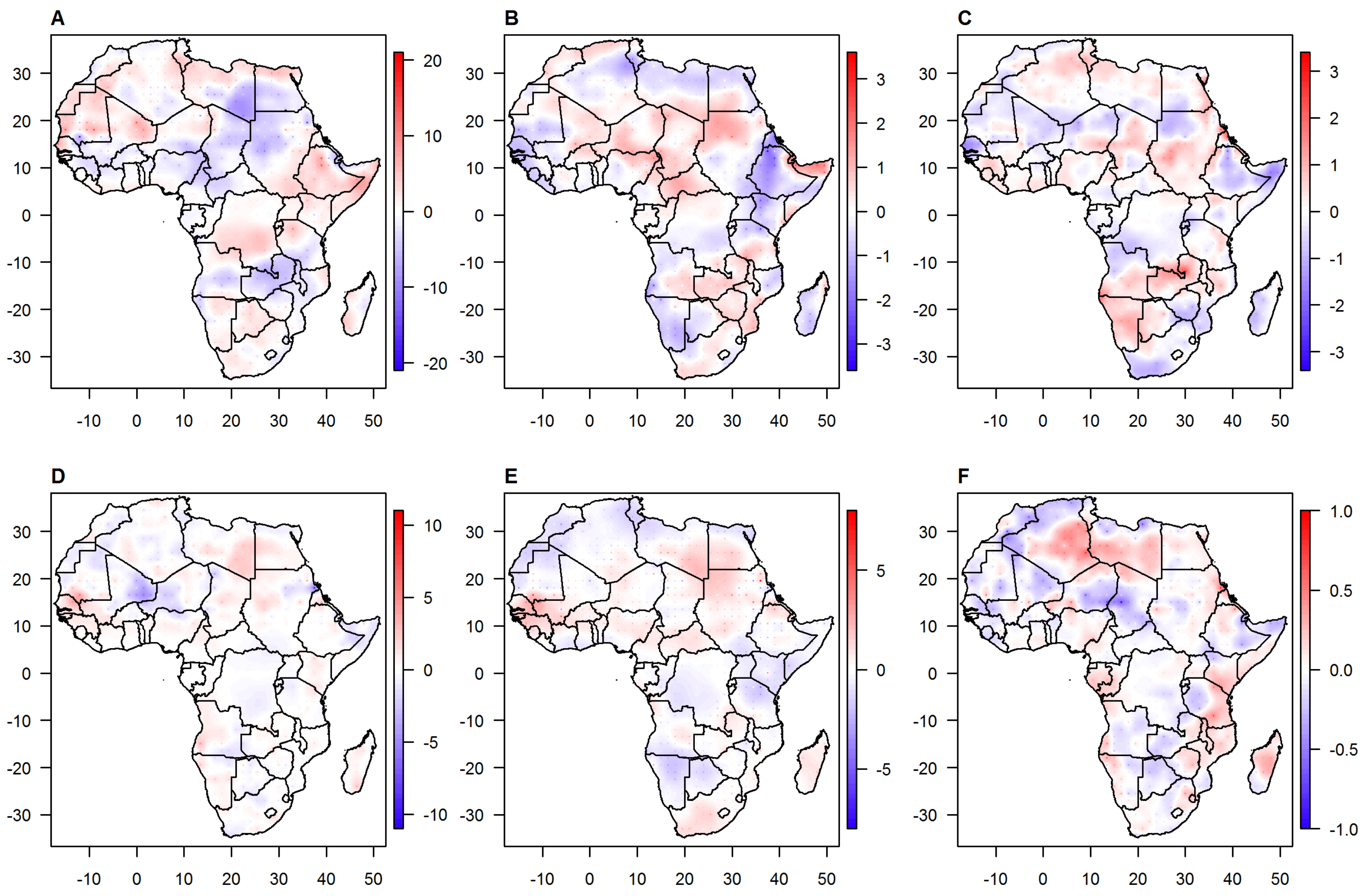

Figure S15: Maps of the random effects for the model fitted to night time air temperatures. Random effects for (A) Intercept, (B) night surface temperature, (C) day surface temperature, (D) EVI, (E) MIR, (F) rainfall. Including pixels of the 1.5° grid on which the model was fitted as well as values interpolated by ordinary kriging to a resolution of 0.1°. The pixels of the original 1.5° grid are slightly increased in size in order to show more clearly the spatial smoothing resulting from the kriging.

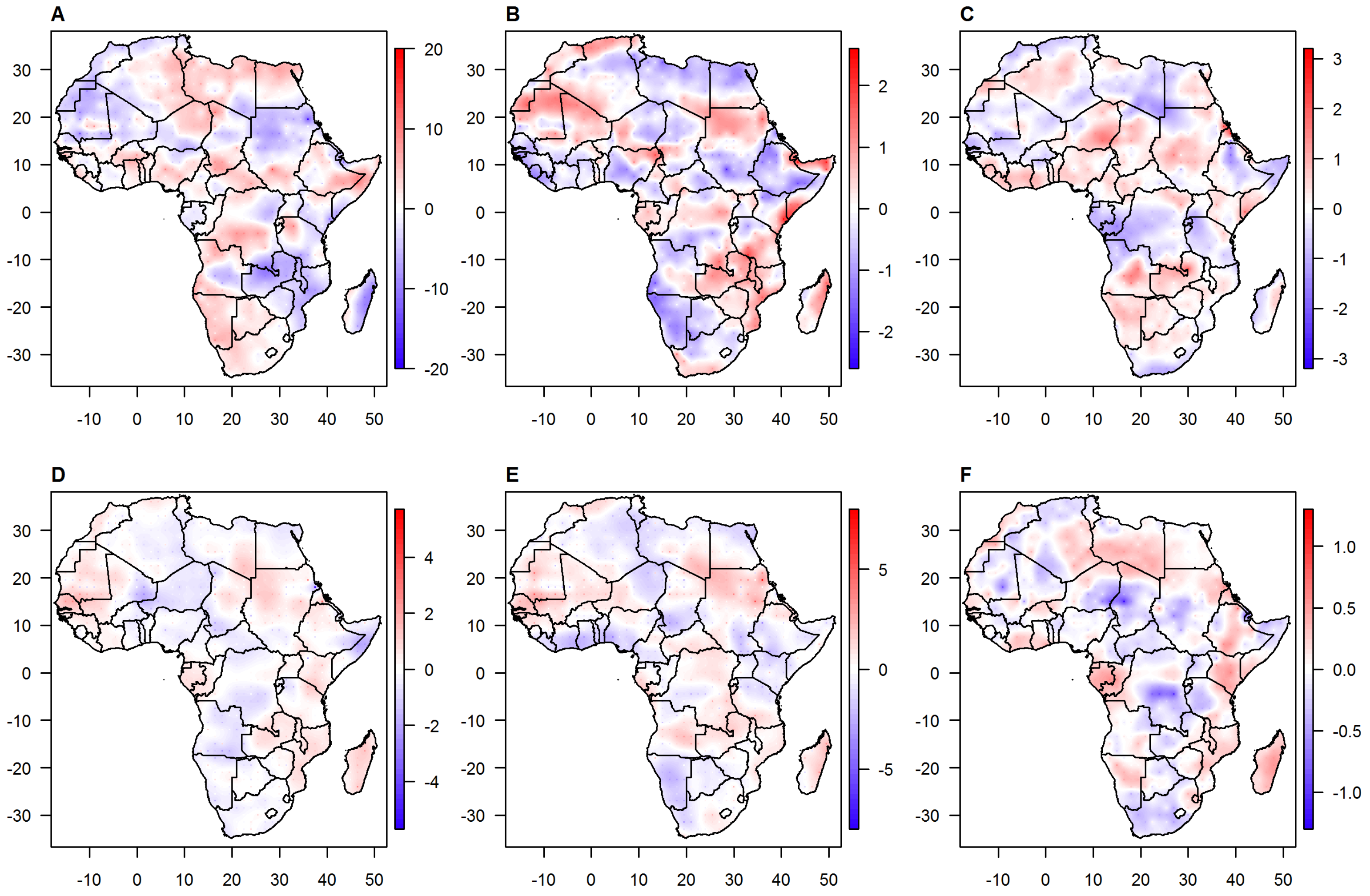

Figure S16: Maps of the random effects for the model fitted to day time air temperatures. Legend as Figure S15, but for the model fitted to day time air temperatures.

References

1. Press WH, Teukolsky SA, Vetterling WT, Flannery BP (1992) Numerical Recipes in C. Cambridge: Cambridge University Press.
2. Isaaks EH, Srivastava RM (1989) Applied Geostatistics. New York: Oxford University Press.
