# Supplementary figures and images for: Estimating Air Temperature and Its Influence on Malaria Transmission across Africa
Source: PLoS One. 2013 Feb 20;8(2):e56487. doi: 10.1371/journal.pone.0056487 (PMC3577915; doi:10.1371/journal.pone.0056487)

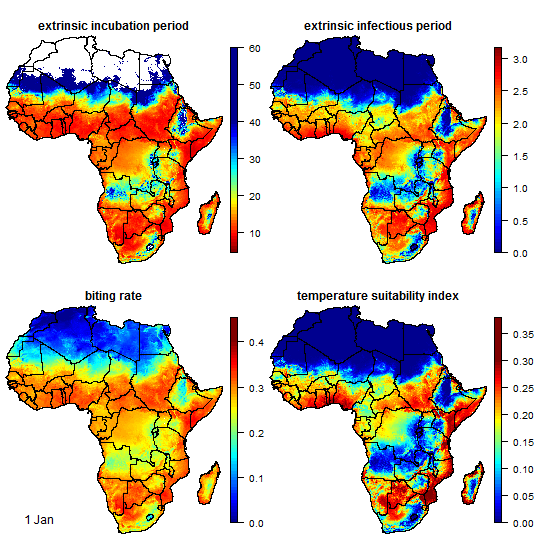

Supplement: Movie S1 — Movie of the extrinsic incubation and infectious periods, the biting rate and the temperature suitability index throughout the year across Africa. (GIF) [file pone.0056487.s002.gif]

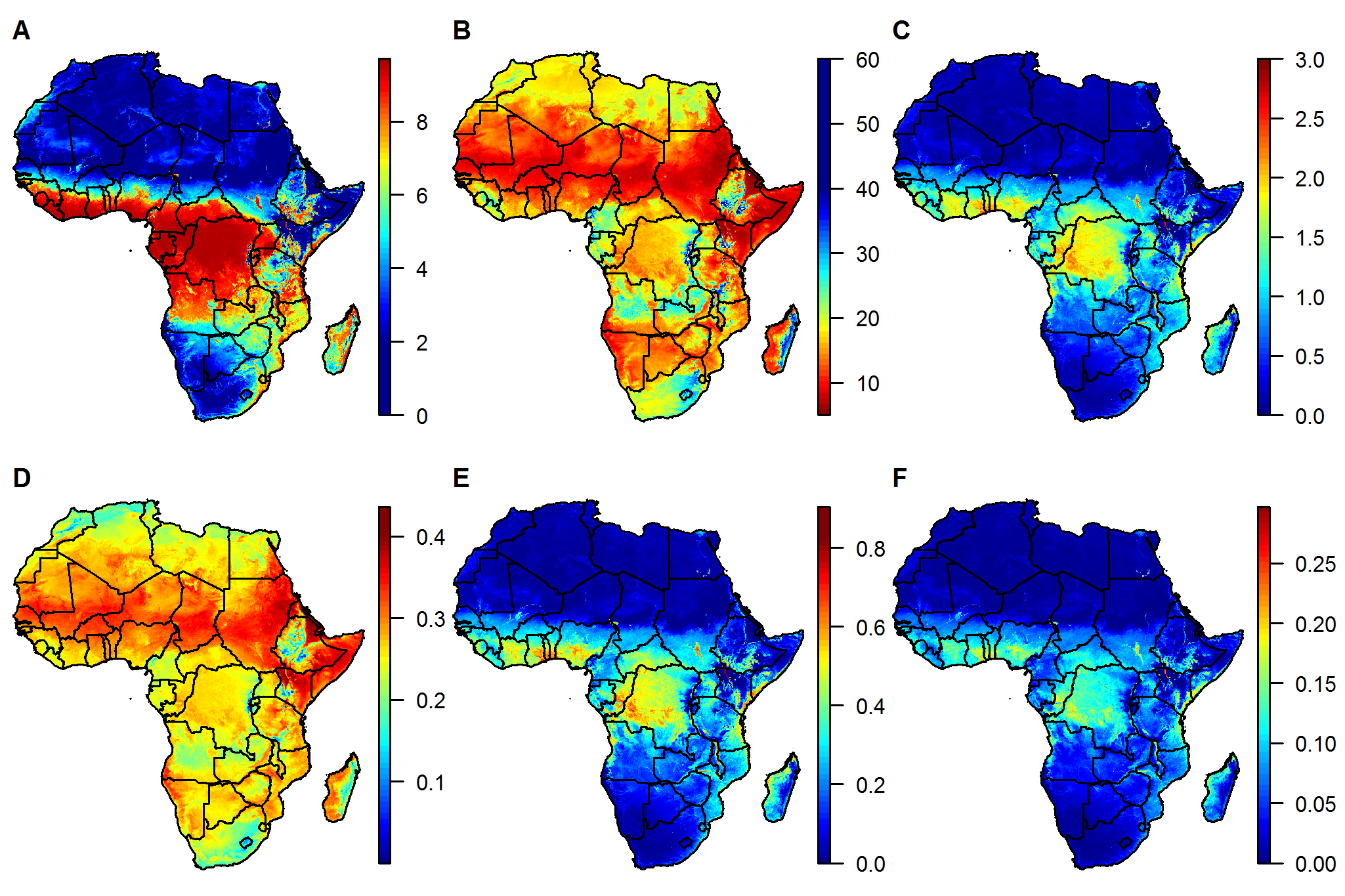

Supplement: File S1 — Maps of the mean annual malaria transmission parameters, calculated using land surface instead of air temperatures as input data. Legend as in Figure 4. (TIF) [file pone.0056487.s003.tif]

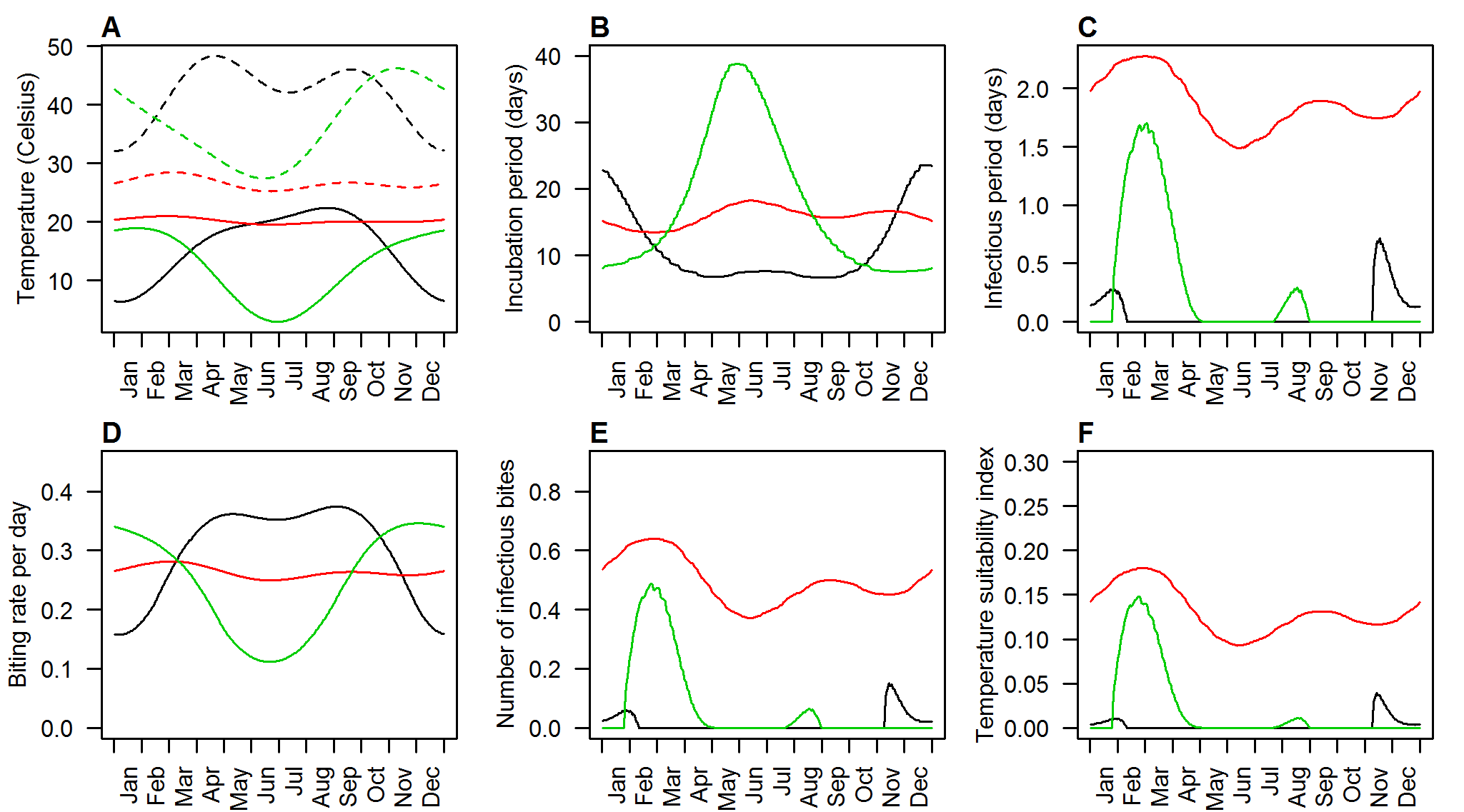

Supplement: File S2 — Seasonality of land surface temperatures as well as several malaria transmission parameters evaluated using land surface instead of air temperatures as input data. Legend as in Figure 5. (TIF) [file pone.0056487.s004.tif]
